# Key genes associated with the comorbidity of allergic rhinitis and chronic rhinosinusitis with nasal polyps: Identification, experimental validation, and an observational study using transcriptomic profiling and Mendelian randomization analysis in humans

**DOI:** 10.1097/MD.0000000000045983

**Published:** 2025-11-28

**Authors:** Xiujuan Hu, Xuemei Wei, De Lan, Zheng Guo

**Affiliations:** a Department of of Otolaryngology, Head, and Neck Surgery, The Affiliated Hospital of Chengdu University, Chengdu, China.

**Keywords:** allergic rhinitis, chronic rhinosinusitis with nasal polyps, key comorbidity genes, Mendelian randomization, pyroptosis

## Abstract

Chronic rhinosinusitis (CRS) with nasal polyps (CRSwNP) frequently coexists with allergic rhinitis (AR). However, the underlying connection between these 2 conditions remains poorly understood. This study aimed to identify and validate key comorbidity genes associated with both AR and CRSwNP, providing novel therapeutic targets and strategies. Transcriptomic data related to AR and CRSwNP were analyzed. Key comorbidity genes were 1st identified through differential expression analysis in the GSE19190 and GSE136825 datasets, Mendelian randomization studies for AR and CRSwNP, and gene expression analysis in AR-related datasets (GSE19190, GSE46171), and CRSwNP-related datasets (GSE136825, GSE179265). Subsequent functional enrichment, immune infiltration, and drug prediction analyses were conducted based on these key comorbidity genes to explore potential therapeutic targets and mechanisms behind the AR–CRSwNP overlap. Reverse transcription quantitative polymerase chain reaction was performed to validate gene expression in clinical samples. Additionally, single-sample gene set enrichment analysis was carried out for CRSwNP. Two key comorbidity genes, CD109 and CPA3, were identified as critical for AR and CRSwNP, with elevated expression in both conditions. Reverse transcription quantitative polymerase chain reaction confirmed a significant increase in CD109 expression in CRSwNP + AR samples (*P* = .01). Functional enrichment analysis revealed that genes linked to AR were primarily associated with “asthma” and “hematopoietic cell lineage,” while CRSwNP-related genes were enriched in “cell cycle” and “allograft rejection.” This study found that significant differences in immune cell infiltration were observed. In the AR group, both CD109 and CPA3 showed the strongest positive correlation with CD56 bright natural killer cells. In the CRSwNP group, CPA3 had the strongest positive correlation with activated B cells, while CD109 was positively correlated with memory CD8 T cells. CRSwNP samples exhibited higher pyroptosis-related gene scores. Furthermore, drug prediction analyses identified 5 potential drugs for CPA3 and 12 for CD109, presenting new treatment avenues for both conditions. CD109 and CPA3 were identified as key comorbidity genes in AR and CRSwNP, with their expression validated in clinical samples. Genes related to pyroptosis may also play a significant role in CRSwNP. These findings provide a theoretical foundation for the development of targeted therapies for AR and CRSwNP.

## 1. Introduction

Allergic rhinitis (AR) is a noninfectious inflammatory disorder mediated by immunoglobulin E (IgE), representing an increasingly significant public health issue worldwide.^[1]^ Common symptoms include nasal itching, sneezing, rhinorrhea, and nasal obstruction.^[2]^ AR frequently coexists with other allergic conditions, such as allergic rhinoconjunctivitis and asthma.^[3]^ Chronic rhinosinusitis (CRS), a heterogeneous disorder characterized by multiple inflammatory endotypes, is not a uniform disease but rather a complex condition with diverse inflammatory subtypes. CRS can be categorized into CRS with nasal polyps (CRSwNP) and CRS without nasal polyps, depending on the presence of polyps.^[4]^ CRSwNP primarily affects the nasal mucosa and paranasal sinuses, driven predominantly by type 2 inflammation.^[5]^ Patients with CRSwNP experience a range of symptoms, including nasal obstruction, rhinorrhea, and impaired olfaction.^[6]^ Individuals with allergies are more susceptible to developing sinusitis, and AR may contribute to the onset or exacerbation of CRSwNP.^[7,8]^ Chronic inflammation is believed to be the underlying mechanism by which AR triggers or worsens CRS.^[9]^ Notably, 56.4% of patients with CRSwNP demonstrate sensitivity to perennial allergens, compared to only 5% in the general population.^[10]^ Given the overlap in inflammatory pathways between AR and CRSwNP, these conditions often coexist.^[11]^ However, the precise pathogenesis linking AR and CRSwNP remains unclear, highlighting the urgent need to investigate their shared molecular and genetic mechanisms to facilitate the development of targeted therapies.

Mendelian randomization (MR) analysis is an epidemiological approach that uses genetic variations as instrumental variables (IVs) to investigate causal relationships between exposure factors (such as traits or diseases) and outcome factors (diseases).^[12]^ This method parallels the random assignment in randomized controlled trials.^[13]^ Since genetic variations are inherited and unaffected by external factors, MR minimizes confounding and reverse causality, making it a powerful tool for causal inference.^[14]^ MR has gained significant recognition for its high level of evidence and has rapidly advanced, emerging as an essential tool for exploring causality. It has also been applied to the study of AR and CRSwNP. For instance, 1 study established a causal link between inflammatory factors and the incidence of AR, CRS, and nasal polyps using MR.^[13]^ Other studies have explored the connection between intestinal microbiota and allergic diseases, suggesting that modulating specific bacterial imbalances could be a novel strategy for preventing and treating AR.^[15]^ Further bioinformatics studies have identified key cell types and genes associated with CRS, leading to the development of diagnostic models and novel therapeutic targets for CRS.^[16,17]^ Additionally, MR has been used to examine the role of AR in cancer development, with 1 study finding no causal relationship between AR and any specific cancers.^[18]^ These findings highlight the potential of MR to elucidate cause-and-effect relationships in complex diseases. Accordingly, this study aims to apply MR analysis to explore the interaction between AR and CRSwNP, identify comorbid genes, and investigate their mechanisms of action, ultimately providing new insights into the development of combined treatments for both conditions.

Based on data from the Gene Expression Omnibus, genome-wide association studies, and Finnish databases, this study employed bioinformatics approaches to identify key comorbidity genes in AR and CRSwNP. Further analyses, including functional enrichment, immune infiltration, regulatory network, and drug prediction analyses, were conducted to elucidate the potential mechanisms of these genes. Notably, the expression of comorbidity genes was validated in clinical samples using reverse transcription quantitative polymerase chain reaction (RT-qPCR). These efforts provide a solid theoretical foundation for the improved diagnosis and treatment of AR and CRSwNP.

## 2. Materials and methods

### 2.1. Data collection

In this study, transcriptome datasets were sourced from the Gene Expression Omnibus database (https://www.ncbi.nlm.nih.gov/geo/). Specifically, Gene Expression Omnibus Series (GSE) 19190 and GSE46171 datasets were associated with AR, while GSE136825 and GSE179265 datasets were linked to CRSwNP. The GSE19190 dataset (GPL6244 platform) included 14 AR nasal airway epithelium tissue samples and 10 control nasal airway epithelium tissue samples, while GSE46171 (GPL6480 platform) contained 6 AR nasal mucosa tissue samples and 3 control nasal mucosa tissue samples. Additionally, the GSE136825 dataset (GPL20301 platform) consisted of 42 CRSwNP nasal polyp tissue samples and 28 control inferior turbinate tissue samples, and the GSE179265 dataset (GPL24676 platform) included 17 CRSwNP nasal polyp tissue samples and 7 control inferior turbinate tissue samples.

Furthermore, the FinnGen database (https://www.finngen.fi/en/access_results) was searched to obtain MR data for both AR and CRSwNP (data downloaded on January 3, 2024). The selection of these datasets was based on the following criteria: the samples were derived from human AR and CRSwNP cases; the data included both case and control groups; and each group contained at least 3 samples. Specifically, the MR data for AR (finngen_R10_ALLERG_RHINITIS) included 444,773 Finns (13,846 cases and 430,927 controls), and the MR data for CRSwNP (finngen_R10_J10_CHRONSINUSITIS) involved 326,444 Finns (308,457 cases and 17,987 controls). Additionally, expression quantitative trait locus data for exposure factors were sourced from the Integrative Epidemiology Unit Open Genome-wide Association Study database (https://gwas.mrcieu.ac.uk/).

### 2.2. Differential expression analysis

In GSE19190, differentially expressed genes 1 (DEGs1) between AR and control samples were identified using the “limma” package (v 3.54.0),^[19]^ with screening criteria set at |log_2_ fold change (FC)| > 0.5 and *P*.adj < .05, with Benjamini–Hochberg correction applied for multiple testing. Similarly, DEGs2 (CRSwNP vs control) in the GSE136825 dataset were identified using the “DESeq2” package (v 3.4.1),^[20]^ with the same screening thresholds (|log_2_FC| > 0.5 and *P*.adj < .05), with Benjamini–Hochberg correction applied for multiple testing. To visualize these 2 sets of DEGs, volcano plots and heatmaps were generated using the “ggplot2” (v 3.3.6)^[21]^ and “complexheatmap” (v 2.15.1)^[22]^ packages, respectively. Notably, the heatmap displayed only the top 10 up- and down-regulated DEGs, ranked by log_2_FC values.

### 2.3. Functional analysis

To further refine the gene list, up-regulated DEGs1 and up-regulated DEGs2, as well as down-regulated DEGs1 and down-regulated DEGs2, were separately intersected using the “venndiagram” package (v 1.7.1),^[23]^ and the results were merged to identify candidate genes. Following this, Gene Ontology (GO) and Kyoto Encyclopedia of Genes and Genomes (KEGG) enrichment analyses were conducted for the candidate genes using the “clusterProfiler” package (v 4.2.2).^[24]^ These analyses aimed to identify the biological functions and signaling pathways associated with the candidate genes, with a significance threshold set at adjusted *P* < .05.

### 2.4. Data pre-processing for MR studies

In this study, 2 separate MR analyses were conducted, with candidate genes treated as exposure factors and AR and CRSwNP as respective outcomes. The classical MR analysis confirmed that the 3 key assumptions were satisfied throughout the analysis: the independence assumption, ensuring that IVs were not associated with confounders; the association assumption, verifying that IVs directly influenced the exposure; and the exclusivity assumption, indicating that IVs could affect the outcome only through the exposure and not via other pathways.

First, the “extract_instruments” function from the “TwoSampleMR” R package (v 0.5.6; MRC Integrative Epidemiology Unit [MRC IEU] at the University of Bristol, Bristol, United Kingdom)^[25]^ was used to extract data for exposure factors and filter IVs (*P* < 5 × 10^−6^). Subsequently, IVs in linkage disequilibrium were removed (*r*^2^ = 0.001, kb = 10, clump = TRUE). The strength of each IV was evaluated using *F* statistics [*F* = *R*^2^ × (N − 2)/(1 − *R*^2^)], with IVs having *F* < 10 being excluded. Next, IVs not associated with the outcome were discarded, while those linked to the exposure factors were retained. Missing values were removed using na.omit(). The effect alleles and effect sizes were then harmonized with the “harmonise_data” function for further analysis.

### 2.5. MR studies and sensitivity analyses

After filtering the IVs, 2 separate MR studies were conducted (one for AR as the outcome and 1 for CRSwNP as the outcome) using the same methods. Five algorithms were applied for the MR analysis: inverse variance weighted (IVW),^[26]^ MR Egger,^[27]^ weighted median,^[28]^ simple mode,^[29]^ and weighted mode,^[28]^ using the ‘mr’ function. Among these methods, IVW was considered the most crucial. The screening criterion for MR analysis was set at *P*_IVW_ < .05. Scatter plots were generated to assess the correlation between the exposure factors and outcomes, while forest plots visualized the diagnostic effectiveness of the exposure factors on the outcomes. Funnel plots were used to examine the symmetry of the causal effect distribution.

Additionally, sensitivity analyses were performed for both MR studies to validate the robustness of the results. These included a heterogeneity test (Cochran *Q* test, *P* > .05) conducted using the “mr heterogeneity” function,^[30]^ assessment of horizontal pleiotropy (*P* > .05) via the “mr pleiotropy test” (*P* > .05)^[31]^ and “mrpresso test” (*P* > .05),^[32]^ and leave-one-out analysis using “mr leaveoneout.”^[33]^ Following this, Steiger analysis was conducted in both MR studies to determine the directionality of the causal relationship using the “steiger_filtering” function.^[34]^ The criteria for passing the Steiger analysis were set to correct causal direction = TRUE and *P* < .05.

Following these comprehensive analyses, core genes1 were identified as having a causal relationship with AR, while core genes2 were found to be causally associated with CRSwNP.

### 2.6. Gene expression analysis

Candidate comorbidity genes were identified by intersecting 2 sets of core genes (core genes1 and core genes2). Gene expression analyses were subsequently performed on these candidate genes using AR-related datasets (GSE19190 and GSE46171) and CRSwNP-related datasets (GSE136825 and GSE179265). Genes exhibiting significant expression differences between AR and control samples (*P* < .05), as well as consistent expression trends across both AR-related datasets, were selected. Similarly, genes showing significant differences between CRSwNP and control samples (*P* < .05) and demonstrating consistent expression patterns across both CRSwNP-related datasets were identified. By integrating these results, key comorbidity genes were selected for further analysis. Additionally, the expression of these genes was validated using a volcano plot generated with the “ggplot2” package (v 3.3.6).

### 2.7. Function analysis of key comorbidity genes

In the AR-related dataset (GSE19190), samples were divided into high- and low-expression groups based on the median expression of each key comorbidity gene. The background gene set used for gene set enrichment analysis (GSEA) was “c2.cp.kegg.v7.5.1.symbols.gmt” from the Molecular Signatures Database (https://www.gsea-msigdb.org/gsea/msigdb). GSEA was performed using the “clusterProfiler” package (v 4.2.2) with the following thresholds: |normalized enrichment score| > 1, nominal *P* < .05, and *q* < .25. A similar GSEA was conducted on the key comorbidity genes in the CRSwNP-related dataset (GSE136825) using the same methods, background gene set, and thresholds.

### 2.8. Immune infiltration analysis

To investigate immune cell infiltration in the AR-related (GSE19190) and CRSwNP-related (GSE136825) datasets, immune infiltration analyses were performed using the single-sample gene set enrichment analysis (ssGSEA) algorithm from the “gene set variation analysis” package (v 4.18.2).^[35]^ This calculated infiltration scores for 28 distinct immune cell types. Differences in infiltration scores between case (AR or CRSwNP) and control groups were assessed using the Wilcoxon test. Differential immune cells (*P* < .05) were identified for further analysis. Subsequently, relationships between differential immune cells and key comorbidity genes were explored using the “corrplot” package (v 0.9.2).^[36]^ This comprehensive analysis was conducted separately in the GSE19190 and GSE136825 datasets, facilitating the exploration of potential interactions between key comorbidity genes and immune cell infiltration patterns in both AR and CRSwNP contexts.

### 2.9. ssGSEA

Necroptosis, induced by tumor necrosis factor-alpha (TNF-α) and interferon gamma (IFN-γ), is known to drive the production of pro-inflammatory cytokines, leading to neutrophil infiltration and exacerbating chronic inflammation in CRSwNP.^[37]^ In the present study, 52 pyroptosis-related genes (PRGs) were selected for ssGSEA analysis (Table S1, Supplemental Digital Content, https://links.lww.com/MD/Q683). The “gene set variation analysis” package (v 1.46.0)^[35]^ was used to compute PRG scores in the GSE136825 dataset based on these 52 genes. The Wilcoxon test was then applied to compare PRG score differences between CRSwNP and control samples, with statistical significance set at *P* < .05.

### 2.10. Regulatory network analysis

To investigate the underlying molecular regulatory network of key comorbidity genes, the microRNA (miRNA) database (https://mirdb.org/) was used to predict miRNAs targeting these genes. Additionally, the iRegulon plugin was employed to identify transcription factors (TFs) that regulate these key comorbidity genes. A miRNA–TF–mRNA network was then constructed and visualized using Cytoscape (v 3.8.2; National Human Genome Research Institute [NHGRI], Bethesda).^[38]^ To identify potential therapeutic drugs for AR and CRSwNP, drugs targeting key comorbidity genes were searched in the Disease Signature Database, and interactions between key comorbidity genes and potential drugs were also visualized using Cytoscape.

### 2.11. RT-qPCR

To validate the expression of key comorbidity genes in clinical samples, 5 CRSwNP + AR samples from patients with both CRSwNP and AR, as well as 5 control samples, were obtained from the affiliated hospital of Chengdu University, with all patients having given their informed consent. Informed consent was obtained from all participants, and the study was approved by the Ethics Committee of the Affiliated Hospital of Chengdu University (approval number: PJ2024-034-03). Total RNA was extracted from the 10 samples using TRIzol reagent (Ambion, USA) according to the manufacturer’s protocol. RNA concentration was assessed using the NanoPhotometer N50. cDNA was synthesized by reverse transcription using the SureScript-First-strand-cDNA-synthesis-kit, and reverse transcription was carried out with the S1000 Thermal Cycler (Bio-Rad, USA). Primer sequences are listed in Table S2, Supplemental Digital Content, https://links.lww.com/MD/Q683. qPCR assays were performed using the CFX connect real-time quantitative fluorescence PCR instrument (Bio-Rad, USA), with the following conditions: pre-denaturation at 95°C for 1 minute, denaturation at 95°C for 20 seconds, annealing at 55°C for 20 seconds, and extension at 72°C for 30 seconds, for a total of 40 cycles. The relative quantification of mRNAs was calculated using the 2^–ΔΔCT^ method.

### 2.12. Statistical analysis

All statistical analyses were performed using R (v 4.2). Differences between 2 groups were compared using the Wilcoxon rank sum test. RT-qPCR experimental results were analyzed using the *t* test. A *P*-value of < .05 was considered statistically significant unless otherwise specified.

## 3. Results

### 3.1. The candidate genes were identified

**Figure 1.**
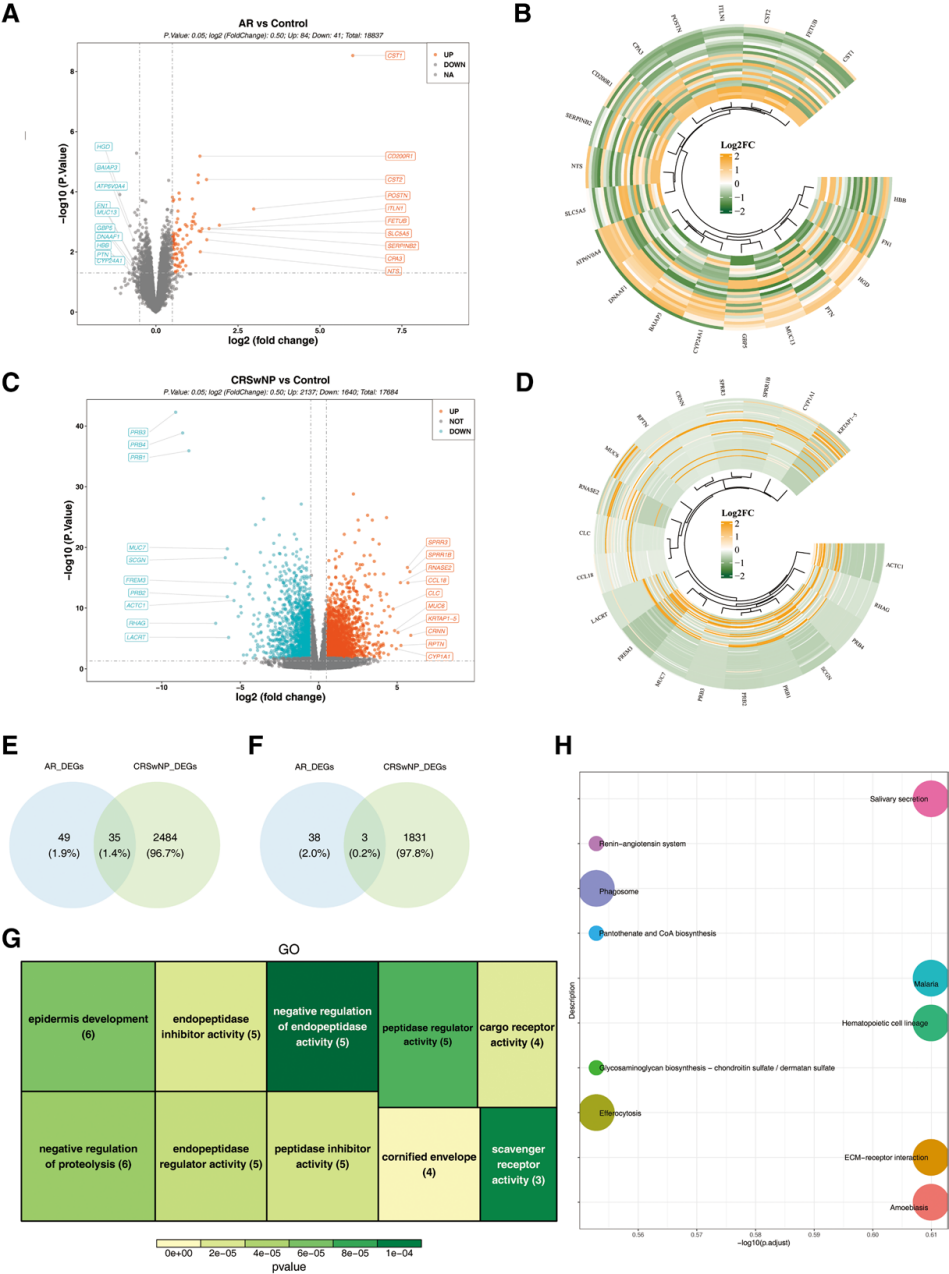
Identification and enrichment pathway of candidate genes in AR and CRSwNP. Volcano plot of differentially expressed genes (DEGs) in AR (A): the horizontal axis is log_2_FC and the vertical axis is −log10(*P*-value); blue for down-regulated genes, red for up-regulated genes, and gray with insignificant differences. Heat map of DEGs in AR (B): the picture is the heat map of top 10 up- and down-regulated genes on the sample. Volcano plot of DEGs in CRSwNP (C). Heat map of DEGs in CRSwNP (D). Venn diagram of comorbidity gene: 35 up-DEG in (E) and 3 down-DEG in (F). Tree diagram of (G): each square represents an entry, the darker the color and the smaller the *P*-value. Enriched bar chart of KEGG (H): the horizontal coordinate is the proportion of genes involved in the KEGG pathway, and the vertical coordinate is the name of the KEGG pathway. The bubble size represents the number of genes involved in the KEGG pathway. AR = allergic rhinitis, CRSwNP = chronic rhinosinusitis with nasal polyps, DEGs = differentially expressed genes, FC = fold change, KEGG = Kyoto Encyclopedia of Genes and Genomes.

In the GSE19190 dataset, a total of 125 DEGs1 were identified, consisting of 84 up-regulated and 41 down-regulated genes in AR samples (Fig. 1A and B). In the GSE136825 dataset, 3777 DEGs2 were identified, including 2137 up-regulated genes and 1640 down-regulated genes in CRSwNP samples (Fig. 1C and D). Subsequently, 38 candidate genes were selected by integrating 35 genes from the overlap of up-regulated DEGs1 and DEGs2, and 3 genes from the overlap of down-regulated DEGs1 and DEGs2 (Fig. 1E and F). Functional analysis revealed significant enrichment of these candidate genes in various GO terms and KEGG pathways. Specifically, the candidate genes were enriched in 42 GO terms, including 21 biological processes (BPs), 3 cellular components (CCs), and 18 molecular functions. Notably, the top 10 GO terms included “negative regulation of endopeptidase activity” (BP), “cornified envelope” (CC), and “endopeptidase regulator activity” (molecular function) (Fig. 1G). Additionally, the 38 candidate genes were associated with 55 KEGG pathways, with the top 10 pathways being “salivary secretion,” “ECM–receptor interaction,” and “phagosome” (Fig. 1H). These findings emphasize the diverse roles of the identified candidate genes in BPs and pathways relevant to AR and CRSwNP, providing a robust foundation for further exploration of their potential roles in disease progression and therapeutic targeting.

### 3.2. The core genes1 and core genes2 had significant causal relationships with AR and CRSwNP, respectively

**Figure 2.**
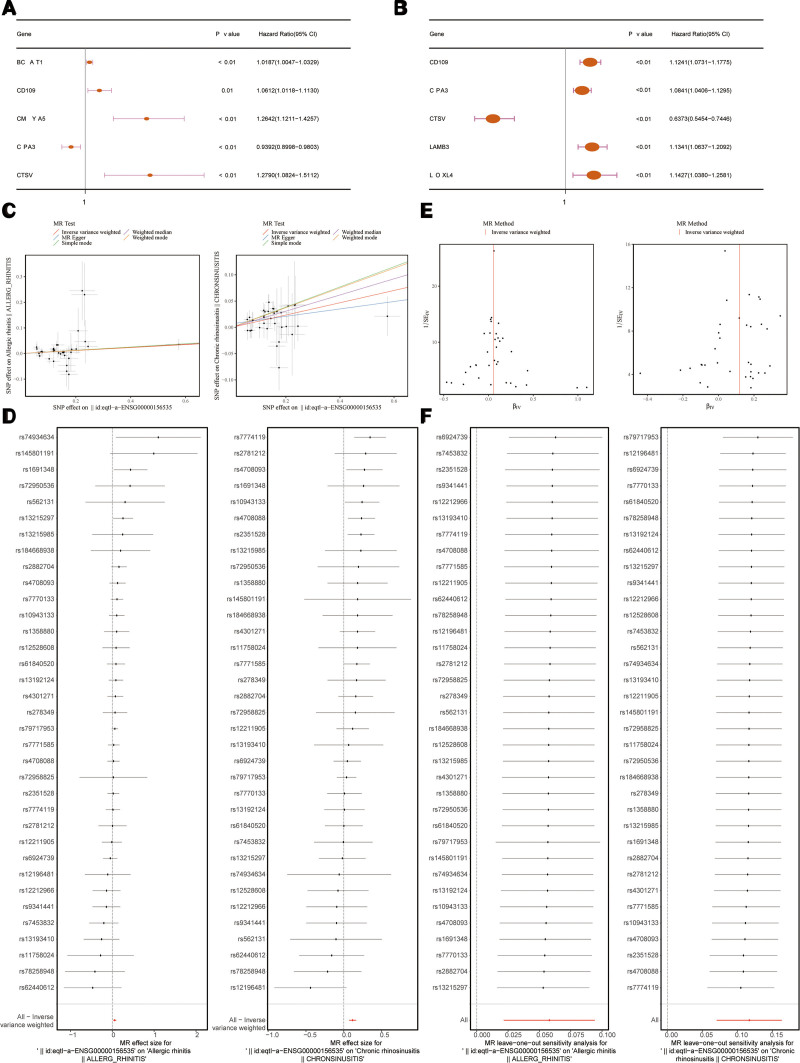
MR analysis of candidate gene in AR and CRSwNP. Forest map of candidate gene in AR (A) and CRSwNP (B) by MR analysis. Scatter plot of SNP effect for CD109 on AR and CRSwNP (C). The horizontal coordinate is the effect of SNP on exposure, the vertical coordinate is the effect of SNP on outcome, and the colored lines represent the fitting results of different MR algorithms. If the slope of a line is positive, it is a risk factor, and if the slope of a line is negative, it is a safety factor. Forest map of SNP for CD109 in AR and CRSwNP (D). SNP solid points on the left mean decrease (safety factor) and on the right mean increase (risk factor). The bottom one is the overall effect of the IVW model. The red dots show a comprehensive estimate by using the IVW method with all SNPS together, and the horizontal lines represent a 95% confidence interval. Funnel map of SNP for CD109 in AR and CRSwNP (E). The horizontal axis is the effect value of the instrumental variable, the vertical axis is the reciprocal of the standard error of the instrumental variable, and the vertical line represents the estimated value of all SNPs. The SNPs are distributed symmetrically, which conforms to the random grouping of Mendel second law. Leave-one-out analysis for CD109 in AR and CRSwNP (F). The effect value of each SNP site on the horizontal axis through exposure factors on the outcome, the vertical axis is the SNP site, the red line is the overall effect value, the black dot represents the IVW estimate after the removal of the SNP, and the red dot represents the IVW estimate of all SNPs. Observe the horizontal deviation position of the black dot relative to the red dot. If the relative position is close, it indicates that the MR result can withstand the test of sensitivity analysis. AR = allergic rhinitis, CRSwNP = chronic rhinosinusitis with nasal polyps, IVW = inverse variance weighted, MR = Mendelian randomization.

After pre-processing the data, extracting exposure factor data, and screening IVs, MR analysis was performed, and the information of the obtained IVs is shown in Table S3 and S4, Supplemental Digital Content, https://links.lww.com/MD/Q683. Among the 38 candidate genes, MR analysis for AR revealed significant causal relationships between 5 core genes1 and AR (*P*_IVW_ < .05). Specifically, CPA3 (odds ratio [OR] = 0.9392, 95% confidence interval [CI] = 0.8998–0.9803, *P* < .01) was identified as a protective factor, while BCAT1 (OR = 1.0187, 95% CI = 1.0047–1.0329, *P* = .01), CD109 (OR = 1.0612, 95% CI = 1.0118–1.1130, *P* = .01), CMYA5 (OR = 1.2642, 95% CI = 1.1211–1.4257, *P* < .01), and CTSV (OR = 1.2790, 95% CI = 1.0824–1.5112, *P* < .01) were associated with increased risk for AR (Fig. 2A). Similarly, MR analysis for CRSwNP identified 5 core genes2 significantly associated with CRSwNP (*P*_IVW_ < .05). Among these, CTSV (OR = 0.6373, 95% CI = 0.5454–0.7446, *P* < .01) was found to be protective, while CD109 (OR = 1.1241, 95% CI = 1.0731–1.1775, *P* < .01), CPA3 (OR = 1.0841, 95% CI = 1.0406–1.1295, *P* < .01), LAMB3 (OR = 1.1341, 95% CI = 1.0637–1.2092, *P* < .01), and LOXL4 (OR = 1.1427, 95% CI = 1.0380–1.2581, *P* = .01) exhibited risk effects for CRSwNP (Fig. 2B). Scatter plots reveal a positive slope for CD109 in both MR studies, indicating a risk association with both AR and CRSwNP (Fig. 2C). The forest plot results indicate that the MR effect size for CD109 was >0 in both studies using the IVW method (Fig. 2D). Funnel plots demonstrate an even distribution of IVs around both sides of the IVW line, supporting the assumption of random assortment according to Mendel second law (Fig. 2E). Additional results, including scatter plots, forest plots, and funnel plots related to AR, are presented in Figures S1 to S3, Supplemental Digital Content, https://links.lww.com/MD/Q681, with corresponding results for CRSwNP shown in Figures S4 to S6, Supplemental Digital Content, https://links.lww.com/MD/Q681.

Furthermore, no heterogeneity was observed (*P* > .05) (Tables 1 and 2). The horizontal pleiotropy analysis showed that in AR, BCAT1, CD109, CMYA5, CPA3, and CTSV had no horizontal pleiotropy; similarly, in CRSwNP, CD109, CPA3, CTSV, LAMB3, and LOXL4 also had no horizontal pleiotropy (Tables 3–6). The leave-one-out analysis detected no outliers, further supporting the reliability of the MR results (Fig. 2F, Figures S7–S8, Supplemental Digital Content, https://links.lww.com/MD/Q681). The Steiger test confirmed that the results of both MR studies were robust and not influenced by reverse causality. In conclusion, these findings reinforce the validity of the MR analyses and their implications for understanding the roles of core genes1 and core genes2 in AR and CRSwNP, respectively.

**Table 1:** The results of heterogeneity test of AR.

Exposure	Outcome	Method	*Q*	*Q*_pval
CD109	AR	MR Egger	31.120	0.740
CD109	AR	Inverse variance weighted	32.094	0.738
CTSV	AR	MR Egger	3.632	0.603
CTSV	AR	Inverse variance weighted	3.844	0.698
CPA3	AR	MR Egger	53.237	0.187
CPA3	AR	Inverse variance weighted	53.899	0.198
CMYA5	AR	MR Egger	0.601	0.996
CMYA5	AR	Inverse variance weighted	0.723	0.998
BCAT1	AR	MR Egger	106.937	0.922
BCAT1	AR	Inverse variance weighted	106.942	0.931

AR = allergic rhinitis, MR = Mendelian randomization.

**Table 2:** The results of heterogeneity test of CRSwNP.

Exposure	Outcome	Method	*Q*	*Q*_pval
CD109	CRSwNP	MR Egger	30.665	.584
CD109	CRSwNP	Inverse variance weighted	31.554	.588
CTSV	CRSwNP	MR Egger	0.383	.603
CTSV	CRSwNP	Inverse variance weighted	0.607	.996
CPA3	CRSwNP	MR Egger	41.750	.482
CPA3	CRSwNP	Inverse variance weighted	43.085	.468
LOXL4	CRSwNP	MR Egger	0.813	.999
LOXL4	CRSwNP	Inverse variance weighted	0.996	.999
LAMB3	CRSwNP	MR Egger	4.084	.995
LAMB3	CRSwNP	Inverse variance weighted	7.387	.946

CRSwNP = chronic rhinosinusitis with nasal polyps, MR = Mendelian randomization.

**Table 3:** The results of horizontal pleiotropy test of AR.

Exposure	Outcome	Egger_intercept	SE	*P*val
CD109	AR	0.007	0.007	.352
CTSV	AR	−0.772	0.168	.665
CPA3	AR	0.005	0.006	.458
CMYA5	AR	−0.021	0.059	.739
BCAT1	AR	−0.0002	0.0003	.940

AR = allergic rhinitis.

**Table 4:** The results of horizontal pleiotropy of CRSwNP.

Exposure	Outcome	Egger_intercept	SE	*P*val
CD109	CRSwNP	-0.0074	0.007	.330
CTSV	CRSwNP	0.0734	0.155	.656
CPA3	CRSwNP	0.0074	0.006	.254
LOXL4	CRSwNP	-0.0114	0.027	.676
LAMB3	CRSwNP	-0.025	0.014	.091

AR = allergic rhinitis, CRSwNP = chronic rhinosinusitis with nasal polyps, MR = Mendelian randomization.

**Table 5:** The results of horizontal MR-PRESSO test of AR.

Exposure	Outcome	*P*val
BCAT1	AR	.439
CD109	AR	.418
CMYA5	AR	.399
CPA3	AR	.424
CTSV	AR	.394

AR = allergic rhinitis, MR = Mendelian randomization.

**Table 6:** The results of horizontal MR-PRESSO test of CRSwNP.

Exposure	Outcome	*P*val
CD109	CRSwNP	.122
CPA3	CRSwNP	.12
CTSV	CRSwNP	.11
LAMB3	CRSwNP	.123
LOXL4	CRSwNP	.156

CRSwNP = chronic rhinosinusitis with nasal polyps, MR = Mendelian randomization.

### 3.3. The CD109 and CPA3 were identified as key comorbidity genes for AR and CRSwNP

**Figure 3.**
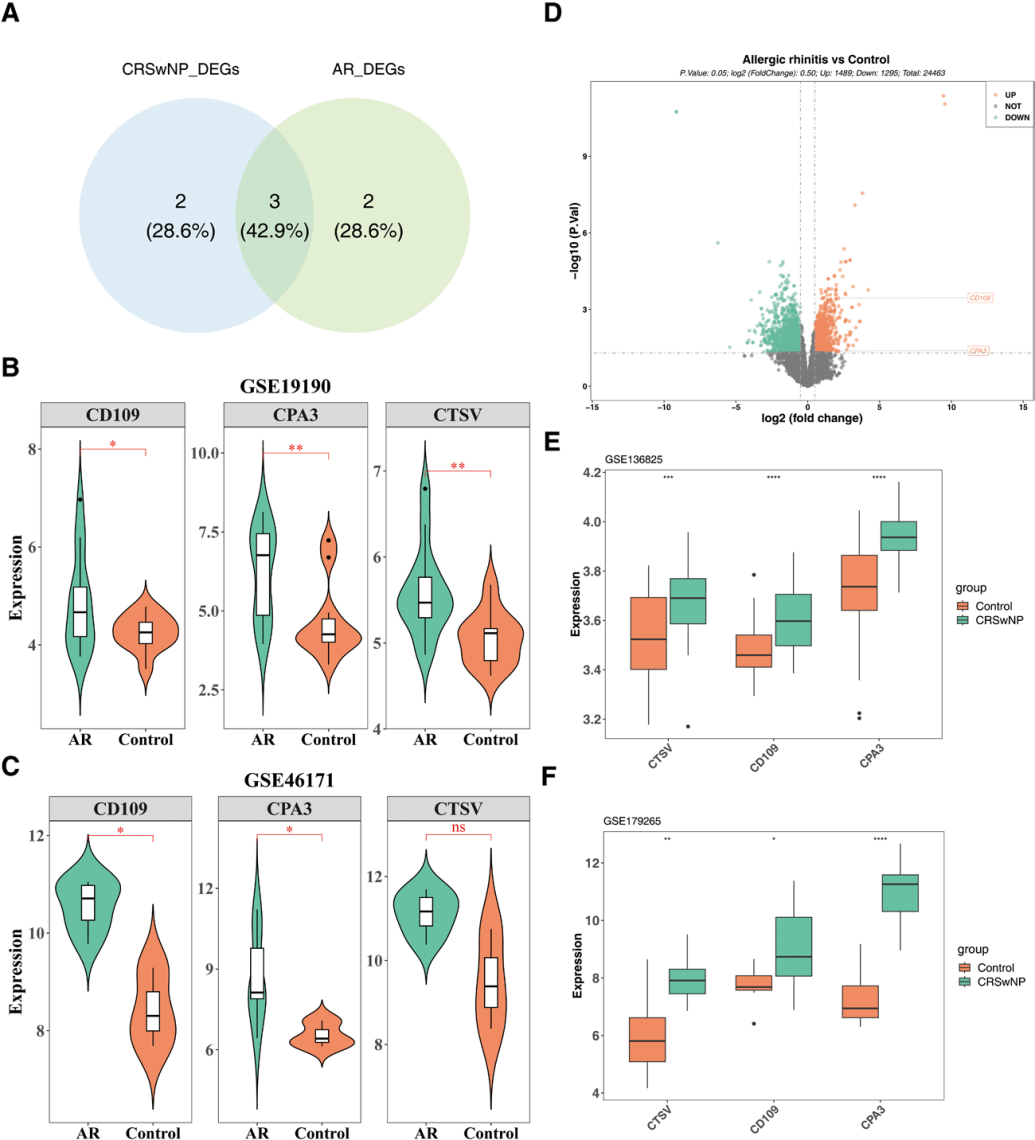
Identification and the expression of comorbidity genes in training and validation sets. Candidate comorbidity gene identification of AR and CRSwNP (A). Expression of candidate comorbidity genes in the AR training set (B) and validation set (C). Volcano map of CD109 and CPA3 expression in the AR validation set (D). Expression validation for key comorbidity genes in the CRSwNP training set (E) and validation set (F). AR = allergic rhinitis, CRSwNP = chronic rhinosinusitis with nasal polyps.

By overlapping core genes1 and core genes2, 3 candidate comorbidity genes (CTSV, CD109, and CPA3) were identified (Fig. 3A). Gene expression analysis of these 3 genes in AR revealed significantly higher expression of CD109 and CPA3 in AR samples across both the GSE19190 and GSE46171 datasets (*P* < .05) (Fig. 3B and C). Validation of CD109 and CPA3 expression was further performed using a volcano plot (Fig. 3D). Similarly, in CRSwNP, gene expression analysis indicated significantly elevated levels of CD109, CPA3, and CTSV in CRSwNP samples across the GSE136825 and GSE179265 datasets (*P* < .05) (Fig. 3E and F). Integrating these findings, CD109 and CPA3 were confirmed as key comorbidity genes for both AR and CRSwNP.

### 3.4. Exploring the function of key comorbidity genes

**Figure 4.**
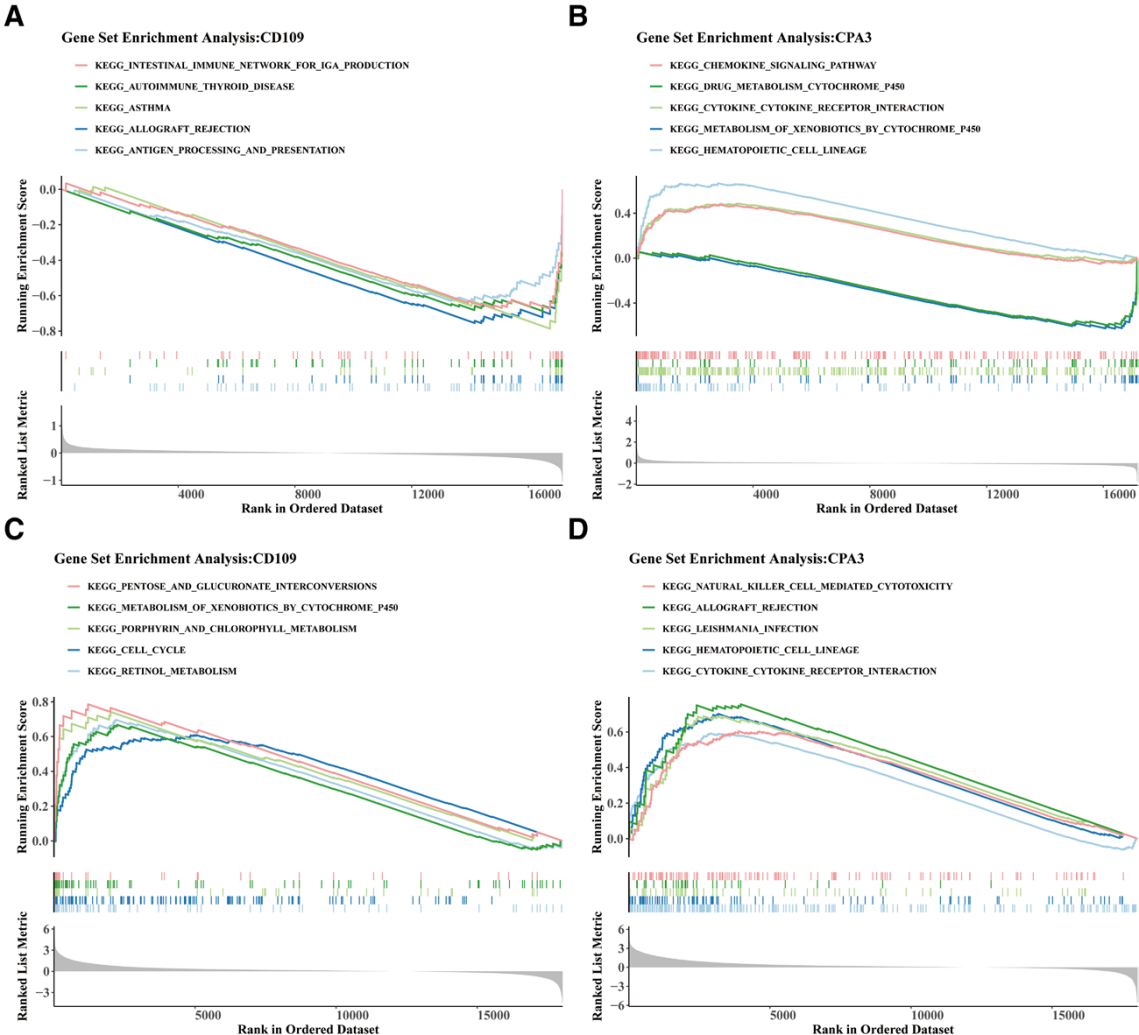
GSEA enrichment analysis of comorbidity genes in AR and CRSwNP. GSEA enrichment analysis of CD109 (A) and CPA3 (B) in AR. GSEA enrichment analysis of CD109 (C) and CPA3 (D) in CRSwNP. AR = allergic rhinitis, CRSwNP = chronic rhinosinusitis with nasal polyps, GSEA = gene set enrichment analysis.

In AR, the top 5 significant KEGG pathways associated with CD109 included “intestinal immune network for immunoglobulin A production,” “autoimmune thyroid disease,” “asthma,” “allograft rejection,” and “antigen processing and presentation” (Fig. 4A). For CPA3 in AR, the top 5 pathways were “chemokine signaling pathway,” “drug metabolism cytochrome P450,” “cytokine–cytokine receptor interaction,” “metabolism of xenobiotics by cytochrome P450,” and “hematopoietic cell lineage” (Fig. 4B). In CRSwNP, the top 5 significant KEGG pathways for CD109 were “pentose and glucuronate interconversions,” “metabolism of xenobiotics by cytochrome P450,” “porphyrin and chlorophyl metabolism,” “cell cycle,” and “retinol metabolism” (Fig. 4C). For CPA3 in CRSwNP, the top 5 pathways included “natural killer cell-mediated cytotoxicity,” “allograft rejection,” “Leishmania infection,” “hematopoietic cell lineage,” and “cytokine–cytokine receptor interaction” (Fig. 4D). These results highlight the distinct pathway associations of CD109 and CPA3 in AR and CRSwNP, suggesting the involvement of different BPs in these conditions.

### 3.5. The differences in immune cell infiltration between case and control samples were explored

**Figure 5.**
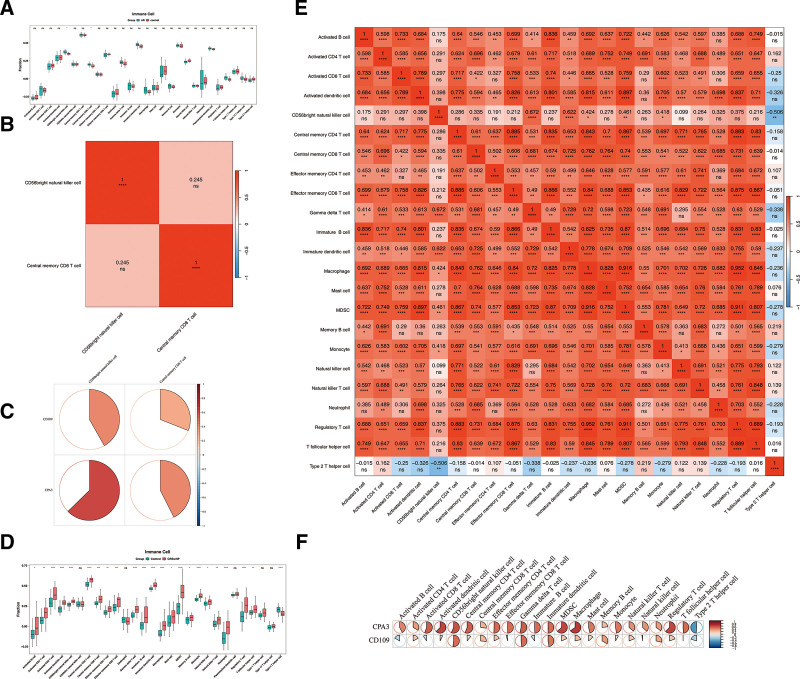
Different immune cells and their relationship with key comorbidity genes. Boxplot of 28 different immune cells in AR and the control group (A). Boxplot of 28 different immune cells in CRSwNP and the control group (D). The correlation between immune cells in AR (B) and CRSwNP (E), which is positive in red and negative in blue. The correlation between key comorbidity genes and immune cells in AR (C) and CRSwNP (F), which show positive correlations in red and negative correlations in blue. AR = allergic rhinitis, CRSwNP = chronic rhinosinusitis with nasal polyps.

Immune infiltration analysis of 28 cell types in AR and control samples from the GSE19190 dataset revealed significant differences in immune cell infiltration for 2 cell types (Fig. 5A). Specifically, CD56 bright natural killer cells and central memory CD8 T cells exhibited significantly higher infiltration scores in AR samples (*P* < .05). Correlation analysis revealed no significant correlation between CD56 bright natural killer cells and central memory CD8 T cells (cor = 0.25, *P* > .05) (Fig. 5B). Further analysis of the correlation between differential immune cells and key comorbidity genes showed that CD109 had a positive correlation with CD56 bright natural killer cells (cor = 0.48, *P* < .05), while CPA3 showed a positive correlation with CD56 bright natural killer cells (cor = 0.63, *P* < .01) and cntral memory CD8 T cells (cor = 0.43, *P* < .05) (Fig. 5C, Table S5, Supplemental Digital Content, https://links.lww.com/MD/Q683). In CRSwNP, significant differences in immune cell infiltration between CRSwNP and control samples were observed for 23 cell types (Fig. 5D). Correlation analysis indicated that CD56 bright natural killer cell exhibited the strongest negative correlation with type 2 T helper cell (cor = −0.51, *P* < .01), while macrophage had the highest positive correlation with regulatory T cell (cor = 0.95, *P* < .01) (Fig. 5E). Additionally, the correlation between differential immune cells and key comorbidity genes showed that CPA3 had a significant positive correlation with activated B cell (cor = 0.42, *P* < .01), natural killer cell (cor = 0.37, *P* < .01), natural killer T cell (cor = 0.36, *P* < .01), neutrophil (cor = 0.35, *P* < .01), activated CD4 T cell (cor = 0.33, *P* < .01), and central memory CD8 T cell (cor = 0.27, *P* < .05), while CD109 demonstrated a positive correlation with memory B cell (cor = 0.33, *P* < .01), neutrophil (cor = 0.29, *P* < .05) and central memory CD8 T cell (cor = 0.27, *P* < .05) (Fig. 5F, Table S6, Supplemental Digital Content, https://links.lww.com/MD/Q683). These results highlight distinct immune cell infiltration patterns in AR and CRSwNP, suggesting that specific immune cell types and their interactions with key comorbidity genes may serve as valuable targets for future therapeutic strategies.

### 3.6. Pyroptosis had an important role in CRSwNP

**Figure 6.**
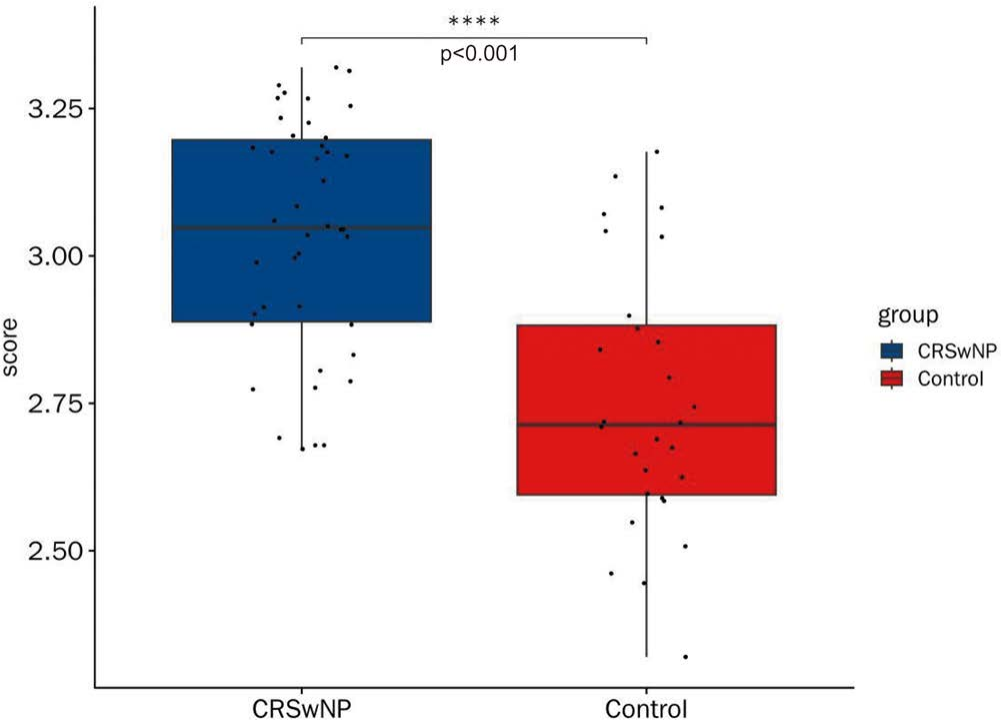
The scores of PRGs in box plot. The scorch death scores in CRSwNP groups is more than in control groups, *P* < .0001. CRSwNP = chronic rhinosinusitis with nasal polyps, PRG = pyroptosis-related gene.

Significant differences in PRG scores were observed between CRSwNP and control samples (*P* < .05) (Fig. 6), with CRSwNP samples exhibiting higher PRG scores. These results suggest that genes associated with pyroptosis may play a critical role in the onset and progression of CRSwNP. The elevated PRG scores in CRSwNP indicate the involvement of pyroptosis-related pathways in the inflammatory processes underlying the condition, highlighting the potential of targeting pyroptosis in the development of therapeutic strategies for CRSwNP.

### 3.7. The regulated network was helpful for exploring the potential mechanism for AR and CRSwNP

**Figure 7.**
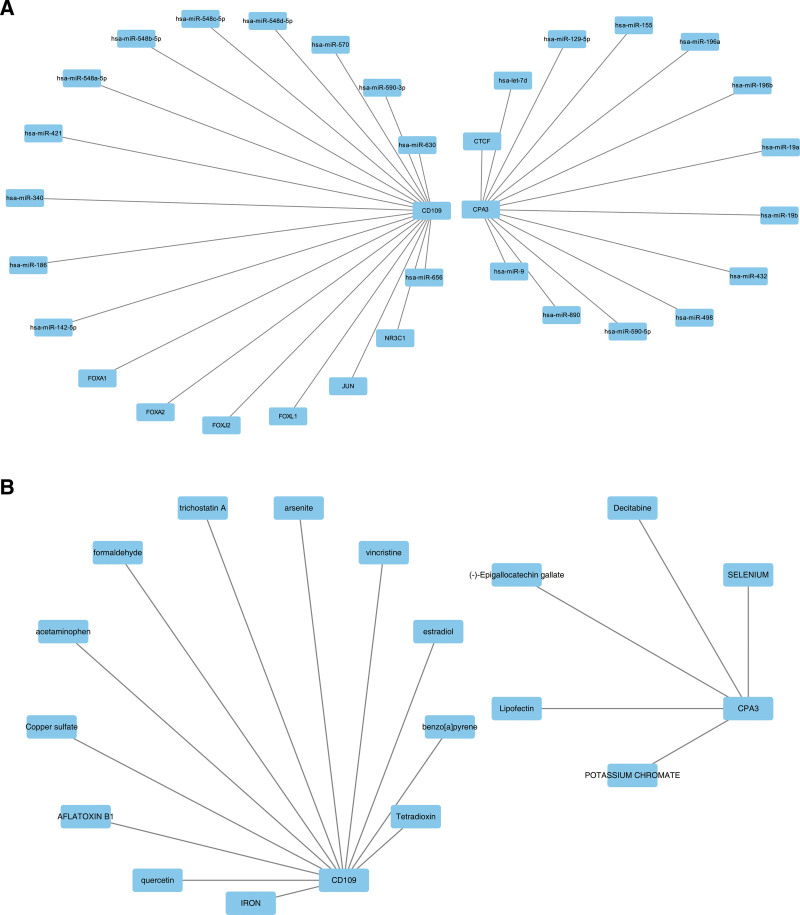
The regulatory networks of key comorbidity gene and the drug prediction. The miRNA–TF–mRNA network diagram (A) includes 33 nodes and 31 edges. Relationship between key comorbidity genes and drugs (B). miRNAs = microRNA, TFs = transcription factors.

From related databases, 12 miRNAs and 1 TF targeting CPA3, and 12 miRNAs and 6 TFs targeting CD109 were predicted, respectively. A miRNA–TF–mRNA network (33 nodes and 31 edges) was constructed based on these miRNAs, TFs, and the 2 key comorbidity genes. Notable relationships in the network included “hsa-miR-630”–CD109–JUN and “hsa-miR-9”–CPA3–CTCF, among others (Fig. 7A). Additionally, 5 drugs targeting CPA3 and 12 drugs targeting CD109 were predicted, and a drugs–key comorbidity genes network was constructed, such as arsenite–CD109 and lipofectin–CPA3 (Fig. 7B). These results highlight potential regulatory mechanisms and therapeutic strategies involving CD109 and CPA3 in AR and CRSwNP, suggesting new avenues for targeted therapies and their associated regulatory networks.

### 3.8. The expression validation of 2 key comorbidity genes

**Figure 8.**
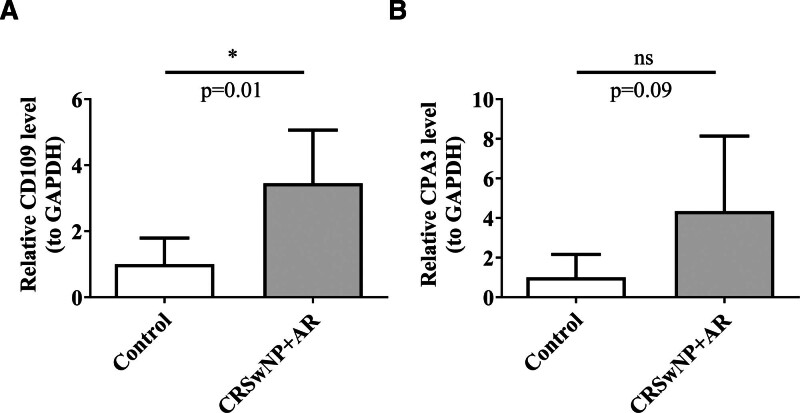
The expression of 2 key comorbidity genes in clinical samples. Relative level of CD109 between CRSwNP + AR and control samples (A). Relative level of CPA3 between CRSwNP + AR and control samples (B). AR = allergic rhinitis, CRSwNP = chronic rhinosinusitis with nasal polyps.

RT-qPCR was employed to validate the expression of the 2 key comorbidity genes in clinical samples. The results showed that CD109 (*P* = .01) was significantly higher in CRSwNP + AR samples, whereas the expression of CPA3 (*P* = .09) did not exhibit a significant difference between CRSwNP + AR and control samples (Fig. 8A and B). These findings underscore the potential role of CD109 as a therapeutic target in patients with both CRSwNP and AR.

## 4. Discussion

CRSwNP often coexists with AR, but the underlying relationship between these 2 conditions remains unclear. This study aimed to identify and validate key comorbidity genes associated with both AR and CRSwNP, thereby providing new therapeutic targets and strategies for treatment. Two key comorbidity genes, CD109 and CPA3, were identified for AR and CRSwNP through transcriptomic data and MR. Both genes exhibited significantly higher expression in AR and CRSwNP samples. RT-qPCR confirmed that CD109 was significantly elevated in samples from patients with both CRSwNP and AR (*P* = .0156). Functional enrichment analysis revealed that the key comorbidity genes in AR were primarily associated with “asthma” and “hematopoietic cell lineage,” while those in CRSwNP were linked to “cell cycle” and “allograft rejection.” Significant differences in immune cell infiltration were observed, with CD109 correlating notably with sebocytes in AR, and both CD109 and CPA3 correlating with adipocytes in CRSwNP. Additionally, potential targeted therapies were identified, with 5 drugs predicted for CPA3 and twelve for CD109, offering new insights into treatment options for AR and CRSwNP. ssGSEA indicated that CRSwNP samples exhibited higher PRG scores. These findings provide a theoretical basis for developing targeted treatments for AR and CRSwNP.

CD109 is a cell membrane glycoprotein that regulates the transforming growth factor β (TGF-β) signaling pathway.^[39]^ Its expression may be elevated in certain inflammatory diseases, influencing disease development by modulating inflammatory and immune responses. CD109 deficiency reduces airway hyperreactivity and eosinophilic inflammation, with decreased levels of Th2 cytokines in mice.^[40]^ CD109 plays a critical role in the pathogenesis of asthma, making it a potential therapeutic target for the disease. Furthermore, CD109 can attenuate epithelial-mesenchymal transition in human nasal epithelial cells by inhibiting the TGF-β1/Smad signaling pathway, thereby suppressing epithelial-mesenchymal transition.^[41]^ Zhang JM et al found that CD109 not only weakened TGF-β1 signaling in human glioblastoma cells but also enhanced EGF signaling.^[42]^ Additionally, CD109 influences the postoperative recurrence of recurrent CRSwNP.^[34]^ The present study further confirms the potential of CD109 as a diagnostic marker for CRSwNP. Similar signaling pathways have been observed in tumor cells, where FLOT2 upregulates CD109 by stabilizing the expression of STAT3, inhibiting the TGF-β signaling pathway and promoting the development of nasopharyngeal carcinoma.^[43]^

CPA3 is an enzyme predominantly expressed in mast cells, responsible for cleaving amino acids from the carboxyl terminus of polypeptide chains, thereby modulating the biological activity of these peptides. This enzyme plays a critical role in regulating peptide activity, which significantly impacts allergic reactions and inflammatory processes. Research has demonstrated that CPA3 is involved in the regulation of specific tissue microenvironments, influencing innate immunity, angiogenesis, and the remodeling of extracellular matrices.^[44]^ In terms of tissue distribution, CPA3 shows the highest protein intensity in skin mast cells and the highest mRNA staining intensity in lung mast cells.^[45]^ Single-cell RNA sequencing of lung mast cells revealed higher CPA3 mRNA expression across all cells.^[46]^ Notably, significantly elevated expression of CPA3 has been observed in mast cells from lung tissue of patients with chronic obstructive pulmonary disease and idiopathic pulmonary fibrosis.^[47]^ Another study identified mast cells in the nasal polyp epithelium of CRSwNP patients expressing tryptase and CPA3 but not chymase. Mast cells that expressed all 3 proteases were abundant in the glandular epithelium of nasal polyps, but not present in normal glandular structures.^[48]^ Through whole-body immune cell screening, CPA3 has been proposed as a potential biomarker for persistent allergic inflammation.^[49]^ Bioinformatics analyses by Yan et al suggested a significant correlation between the CPA3 gene and novel biomarkers related to rhinitis and asthma complications.^[50]^ Mast cells, primarily through the release of CPA3, are implicated in the pathological processes of AR, positioning CPA3 as a promising diagnostic marker for the condition.^[51]^ This study represents the first identification of the correlation between CD109 and CPA3 genes and the complications of AR and CRSwNP.

RT-qPCR offers advantages such as high specificity, sensitivity, simplicity, speed, and low DNA purity requirements. In this study, RT-qPCR was used to detect gene expression levels in clinical samples, revealing expression differences under various conditions and confirming the consistency of the MR analysis results through experimental verification. The expression of 2 key comorbidity genes, CD109 and CPA3, was verified in clinical samples, with RT-qPCR results showing that CD109 was significantly overexpressed in CRSwNP + AR patients, while CPA3 was also overexpressed, but without statistical significance. This may be due to CD109 acting as a common risk factor for both AR and CRSwNP. Previous studies have shown that blocking CD109 with monoclonal antibodies can inhibit airway inflammation in a house dust mite-induced asthma mouse model. The mechanism underlying CD109’s effect on allergic airway inflammation in dendritic cells (DCs) likely involves regulation of the TGF-β cytokine, which inhibits T cell proliferation and induces Treg differentiation. CD109 expression on T cells may amplify Th2 effector function by inhibiting TβR signal transduction.^[52]^ Since CRSwNP is predominantly driven by type 2 inflammation^[2]^ and AR inflammation is also primarily mediated by Th2 cells,^[53]^ both conditions share similar pathogenic mechanisms related to airway allergic inflammation. Therefore, the high expression of CD109 may influence the inflammatory response in CRSwNP and the immune response in AR by regulating the TGF-β signaling pathway.^[42]^ However, the specific regulatory mechanisms remain to be further explored. CPA3 is localized primarily in the nasal mucosa interstitium in AR, and its upregulation has been confirmed in AR patients.^[43]^ The expression patterns of CD109 and CPA3 observed in this study through RT-qPCR align with previous findings.

A comprehensive analysis of key comorbidity genes related to immune cell infiltration in AR has revealed that immune-related DEGs are associated with T cell activation, leukocyte cell–cell adhesion, and cytokine–cytokine receptor interactions. T cell receptor signaling pathways are notably altered or activated in AR, and dysregulation of these processes contributes to immune responses and the development of AR.^[54]^ Furthermore, cytokine–cytokine receptor interactions have been enriched in CRSwNP,^[55]^ suggesting that this pathway activates immune cells and promotes the release of inflammatory mediators, which exacerbates symptoms and contributes to CRSwNP formation. Previous research has also highlighted the significance of “cell adhesion molecule binding,” “cytokine receptor binding,” and “glucocorticoid receptor binding” in eosinophilic CRSwNP.^[56]^ The 3.4 GSEA enrichment analysis in this study revealed that CPA3 is enriched in both AR and CRSwNP within the cell–cytokine receptor and hematopoietic cell lineage pathways, suggesting that CPA3 influences the pathogenesis of AR and CRSwNP through shared signaling mechanisms. In contrast, CD109 affects the pathogenesis of AR and CRSwNP through distinct pathways, which aligns with previous findings.

This study found that the infiltration scores of CD56 bright natural killer cells and central memory CD8 T cells were both high in AR and CRSwNP samples. Two significantly different infiltrating immune cells were observed in the AR group, while 23 significantly different infiltrating immune cells were found in the CRSwNP group. In the AR group, both CD109 and CPA3 showed the strongest positive correlation with CD56 bright natural killer cells. In the CRSwNP group, CPA3 had the strongest positive correlation with activated B cells; CD109 was positively correlated with memory CD8 T cells.

CD56 bright natural killer cells are a subset of natural killer cells,^[57]^ secreting pro-inflammatory cytokines such as IFN-γ or immunosuppressive TGFβ and interleukin-10 (IL-10), while performing cytotoxicity and participating in immune regulation.^[58]^ Both AR and CRSwNP are chronic type 2 inflammatory diseases (especially the eosinophilic CRSwNP), involving Th2 cytokines (IL-4, IL-5, IL-13), eosinophilic infiltration and tissue remodeling. IFN-γ theoretically can inhibit Th2 responses and thereby control the Th1/Th2 balance.^[59]^ However, in a strong Th2 microenvironment, its function may be suppressed or shift towards the production of Th2 cytokines (such as IL-13), thereby exacerbating inflammation and mucus secretion. In Th2-dominant polyps, the situation may be similar to AR (suppression or pro-inflammatory shift). Central memory CD8 T cells can initiate and maintain allergic inflammation through T cells, eosinophils and innate lymphoid cells 2; promote IgE class switching; and open the epithelial barrier.^[60]^ Research has found that the CD4/CD8 ratio is decreased in patients with AR, the proportion of memory CD4 + T cell subsets is increased, Th2 response is imbalanced, and CD8 + T cell activation is enhanced.^[61]^ In addition, T lymphocytes are commonly present in the sinus mucosa and are related to the pathogenesis of CRS, and the percentage of memory CD8 + T cells is the highest in mucosa of CRSwNP.^[62]^ Activated B cells and the antibodies they produce (especially IgE) are important amplifiers of type 2 inflammation. Both AR and CRSwNP have evidence of pathological type 2 inflammation, B cell activation and differentiation, and IgE production. The B cell activation in AR may occur more frequently in the germinal centers of lymph follicles for regulation, while CRSwNP may occur through extranodal pathways.^[63]^ The T cell immunoglobulin mucin domain 4 + B cells in the nasal mucosa of AR can induce Th2 polarization. B cells play an important role in the initiation of Th2 polarization.^[64]^ In CRSwNP, B cell activation factors induce the production of Immunoglobulin A, causing eosinophil aggregation and degranulation, thereby leading to aggravated tissue inflammation and ultimately the formation of polyps.^[65]^ The immune cell infiltration patterns significantly influence disease development by modulating the inflammatory process, determining disease phenotype, and participating in the underlying disease mechanisms. The distinct roles of specific immune cell types and key comorbidity genes in AR and CRSwNP present promising targets for future therapeutic strategies.

Immune cells play a critical role in the human immune system, directly influencing the occurrence, progression, and defense against diseases. During the early stages of CRSwNP, antigen-presenting cells, such as DCs, are pivotal.^[66,67]^ Additionally, the imbalance of Th cells, particularly the dysregulation between Th1 and Th2 cells, has been extensively studied. Macrophage pyroptosis, mediated by NOD-like receptor protein 3 (NLRP3), is known to influence Th1/Th2 differentiation.^[68]^ As a pro-inflammatory mechanism, pyroptosis significantly impacts the autoimmune response associated with the disease. The activation of the NLRP3 inflammasome triggers the release of inflammatory cytokines, such as IL-1β and IL-18, through crucial proteins like caspase-1 and gasdermin D. These processes disrupt intracellular homeostasis and exacerbate inflammation.^[69]^ Recent studies have identified key PRGs (AIM2, CASP5, and NLRP6) that are closely linked to the onset and progression of CRSwNP. This highlights the pivotal role of pyroptosis in CRSwNP, supporting the exploration of potential biomarkers and therapeutic targets for the disease.^[70]^

Moreover, the critical role of gut microbiota has been increasingly recognized in various diseases. Probiotics, prebiotics, and commensal bacteria influence the gut microbiota composition in AR by counteracting harmful bacteria and regulating host metabolism. Bacterial lysates affect the gut environment indirectly by stimulating DCs within the intestinal mucosa, modulating immune responses, and reducing the secretion of antigen-specific IgE and Th2 cytokines (IL-4, IL-13).^[71]^ Further research in these areas could provide valuable insights for developing new therapeutic strategies.

Regulatory network analyses predicted 5 potential drugs targeting CPA3 and 12 potential drugs targeting CD109. Some studies have suggested that selenium (Se) and vitamin E, known for their immunomodulatory and antioxidant effects, could serve as adjunctive treatments. When combined, vitamin E and Se can help control allergic mediators, alleviate symptoms associated with rhinitis and asthma, reduce airway mucus secretion, and assist in the dilation of obstructed bronchi.^[72]^ Additionally, the use of a multivitamin-mineral supplement containing Se has been proposed as adjunctive therapy for children with chronic or recurrent sinusitis.^[73]^ Based on our findings, Se–CPA3 may represent a potential therapeutic approach for treating both AR and CRSwNP. Quercetin, a plant-derived flavonoid, exhibits various pharmacological effects.^[74]^ It may be beneficial for AR treatment by balancing the Th1/Th2 and Treg/Th17 ratios and inhibiting the NF-κB pathway.^[75]^ Furthermore, quercetin has been shown to reduce cell apoptosis and attenuate CRS progression by inhibiting nasal mucosal inflammation and epithelial barrier dysfunction, thus supporting its potential as a therapeutic agent for CRS.^[76]^ These findings align with our research on the quercetin–CD109 interaction. It should be noted that although Se and quercetin have shown therapeutic potential, this conclusion is based on preliminary research results and inferences from existing literature, which may affect the reliability of the conclusion and requires experimental verification. These predictions have not been fully validated in extensive clinical practice, so their transformation into clinical applications still needs to go through strict scientific verification. Thorough preclinical and clinical studies must be carried out to ensure their safety and efficacy and to avoid premature or unreasonable application in clinical practice. In addition, further research is needed on their bioavailability, the best administration route, and potential interactions with other drugs in clinical practice.

Although this study explored the relationship between AR and CRSwNP through multiple bioinformatics methods, providing new ideas for our future exploration of related diseases and drugs in this direction, it also has some limitations. Firstly, this study mainly relied on bioinformatics analysis and lacked direct experimental verification. Secondly, the acquisition of clinical samples for RT-qPCR was difficult, and the number used in the experiment was too small, which may affect the statistical power and the reliability of the conclusion. Although MR can provide valuable clues about causal associations, it cannot directly prove causality. In the future, we will attempt to obtain more clinical samples, expand the sample cohort size to enhance the persuasiveness of the RT-qPCR experimental results, and further explore and verify the regulatory mechanisms of CD109 and CPA3 through additional experimental methods such as gene knockout, gene overexpression, or pathway-specific detection. Moreover, we will combine prospective cohort studies with molecular-level mechanism research to confirm whether the causal relationship identified by this MR analysis is truly existent.

This study identified 2 key comorbidity genes associated with AR and CRSwNP through transcriptomic and MR analyses. Additionally, GSEA, immune infiltration analysis, regulatory network analysis, and drug prediction were conducted based on these genes. These results may offer new insights into the clinical diagnosis and treatment of AR and CRSwNP. By identifying comorbidity genes, this study aims to address the issue of AR and CRSwNP comorbidities in patients with a single class of drugs, thus reducing the drug burden. RT-qPCR validation was also performed to analyze the expression of key comorbidity genes in clinical samples, providing a foundation for considering these genes as potential therapeutic targets.

## Acknowledgments

We would like to express our sincere gratitude to all individuals and organizations who supported and assisted us throughout this research. We extend our thanks to everyone who has supported and assisted us along the way. Without your support, this research would not have been possible. And thanks for the supporting of Chengdu Medical Research Project and the research project of Affiliated Hospital of Chengdu University.

## Author contributions

**Conceptualization:** Xiujuan Hu.

**Formal analysis:** Xiujuan Hu, Zheng Guo.

**Investigation:** Xiujuan Hu, Zheng Guo.

**Project administration:** Xiujuan Hu, Xuemei Wei.

**Resources:** Xiujuan Hu, De Lan, Zheng Guo.

**Writing – original draft:** Xiujuan Hu.

**Writing – review & editing:** Xuemei Wei, De Lan.

## Supplementary Material




