# Supplementary material for: Key genes associated with the comorbidity of allergic rhinitis and chronic rhinosinusitis with nasal polyps: Identification, experimental validation, and an observational study using transcriptomic profiling and Mendelian randomization analysis in humans
Source: Medicine (Baltimore). 2025 Nov 28;104(48):e45983. doi: 10.1097/MD.0000000000045983 (PMC12662326; doi:10.1097/MD.0000000000045983)
Supplement: Supplementary file 1 [file medi-104-e45983-s001.pdf]

Table S1 The 52 PRGs.

No.	PRGs
1	BAK1
2	BAX
3	CASP1
4	CASP3
5	CASP4
6	CASP5
7	CASP6
8	CASP8
9	CASP9
10	CHMP2A
11	CHMP2B
12	CHMP3
13	CHMP4A
14	CHMP4B
15	CHMP4C
16	CHMP6
17	CHMP7
18	CYCS
19	ELANE
20	GPX4
21	GSDMB
22	GSDMC
23	GSDMD
24	GSDME
25	GZMB
26	HMGB1
27	IL18
28	IL1A
29	IL1B
30	IRF1
31	IRF2
32	NLRC4
33	NLRP1
34	NLRP2
35	NLRP3
36	NLRP6
37	NLRP7
38	NOD1
39	PLCG1
40	PJVK

41	PRKACA
42	PYCARD
43	SCAF11
44	TINAP
45	TNF
46	TP53
47	TP63
48	AIM2
49	GSDMA
50	IL6
51	NOD2
52	TIRAP

Table S2 The sequences and all primers in RT-qPCR

Primer	Sequence
CD109 F	TCTGGAACACTGCCCTTCAC
CD109 R	TTGGGGTCTGATGGAAGAGT
CPA3 F	TGACCTGGGCATCAAACACA
CPA3 R	TAGCATGGTCTCTCTGCACG
Internal reference-GAPDH F	CGAAGGTGGAGTCAACGGATTT
Internal reference-GAPDH R	ATGGGTGGAATCATATTGGAAC

Table S3 AR tool variable information

SNP	id	pvalue	r2	F
rs10743523	eqtl-a-ENSG00000060982	1e-200	0.0732446331440674	2494.13622561714
rs10771146	eqtl-a-ENSG00000060982	4.25e-117	0.0170904204828417	529.121494541582
rs10842462	eqtl-a-ENSG00000060982	3.13e-21	0.00282561292023563	89.4488709385026
rs10842509	eqtl-a-ENSG00000060982	2.73e-19	0.00254752945088961	80.6155901780591
rs10842523	eqtl-a-ENSG00000060982	1.46e-08	0.00102475210715005	32.1055917223509
rs10842531	eqtl-a-ENSG00000060982	2.23e-07	0.000857860669897878	26.8208601673403
rs11047386	eqtl-a-ENSG00000060982	2.19e-06	0.000709773348112755	22.4156471219983
rs11047431	eqtl-a-ENSG00000060982	3.11e-14	0.00195112777685608	57.6629727339443
rs11047478	eqtl-a-ENSG00000060982	1.41e-17	0.00465844597794997	72.8246692042516
rs11047548	eqtl-a-ENSG00000060982	1.63e-07	0.000918547483886134	27.4291406023444
rs11047573	eqtl-a-ENSG00000060982	1.25e-38	0.00618887998466657	168.956152212704
rs11047735	eqtl-a-ENSG00000060982	5.7e-106	0.0148629243779822	477.991521987855
rs11047769	eqtl-a-ENSG00000060982	4.97e-18	0.002483635337621	74.8813106482047
rs11047836	eqtl-a-ENSG00000060982	5.62e-10	0.00542900278847087	38.4397270216002
rs11048077	eqtl-a-ENSG00000060982	6.2e-07	0.0007866791879343	24.8518656918552
rs111985454	eqtl-a-ENSG00000060982	4.63e-32	0.00447269110771518	138.88998490434
rs112509460	eqtl-a-ENSG00000060982	1.12e-11	0.00166316097909394	46.1013277037878
rs113082066	eqtl-a-ENSG00000060982	7.82e-13	0.00207195337669153	51.3250305421551
rs113824096	eqtl-a-ENSG00000060982	2.51e-12	0.00199163511543358	49.0381157248215
rs113934831	eqtl-a-ENSG00000060982	3.3e-08	0.00788232194096608	30.5085948196447
rs116459693	eqtl-a-ENSG00000060982	2.3e-06	0.000714286487215803	22.3223972960131

rs11650106	eqtl-a-ENSG00000060982	1.56e-06	0.00072771982799129	23.0724098405406
rs116976358	eqtl-a-ENSG00000060982	3.33e-79	0.0176192511419235	355.04634634886
rs116979072	eqtl-a-ENSG00000060982	7.91e-16	0.0021554078311062	64.8904720859596
rs117016831	eqtl-a-ENSG00000060982	1.46e-07	0.00105357057489505	27.6358260671322
rs117112720	eqtl-a-ENSG00000060982	8.04e-07	0.000778953523055304	24.3488009233708
rs117203103	eqtl-a-ENSG00000060982	2.96e-12	0.00643441612275652	48.7066424648127
rs117218709	eqtl-a-ENSG00000060982	2.16e-12	0.0198682893650679	49.299169909056
rs117280971	eqtl-a-ENSG00000060982	1.05e-26	0.0101117920507667	114.419165569499
rs117291368	eqtl-a-ENSG00000060982	1.6e-90	0.0218629910911643	407.02382605299
rs117312229	eqtl-a-ENSG00000060982	7.71e-18	0.0028729726230048	74.016440920788
rs117395935	eqtl-a-ENSG00000060982	6.71e-19	0.00369571718148282	78.8401438494508
rs117642638	eqtl-a-ENSG00000060982	4.45e-14	0.00271905015703811	56.9558235214163
rs117791138	eqtl-a-ENSG00000060982	5.65e-67	0.0104811910791714	298.901575590769
rs117922518	eqtl-a-ENSG00000060982	4.17e-06	0.000711213673128898	21.1850532939234
rs117987067	eqtl-a-ENSG00000060982	2.35e-06	0.000740498679125227	22.2870012696931
rs118138049	eqtl-a-ENSG00000060982	1.01e-16	0.00441164482851028	68.9493736794419
rs12301149	eqtl-a-ENSG00000060982	1e-200	0.0748383809730483	2250.42167319908
rs12302883	eqtl-a-ENSG00000060982	1e-200	0.0377525159651935	1243.00165046313
rs12315511	eqtl-a-ENSG00000060982	1.26e-10	0.00130836466264803	41.3539449814226
rs12368110	eqtl-a-ENSG00000060982	8.48e-10	0.00119137027174269	37.6421087249564
rs12369764	eqtl-a-ENSG00000060982	1.47e-06	0.000794614638914105	23.1790512939037
rs12370682	eqtl-a-ENSG00000060982	2.53e-10	0.00681762613185829	39.9990057370999

rs12423047	eqtl-a-ENSG00000060982	1.53e-20	0.00272701277862926	86.3190054137918
rs12423264	eqtl-a-ENSG00000060982	6.15e-28	0.00377473515189261	120.044294500497
rs12423489	eqtl-a-ENSG00000060982	2.88e-19	0.00254398275451581	80.5107214988474
rs12425968	eqtl-a-ENSG00000060982	3.03e-10	0.00130488589444508	39.6550321885549
rs12426684	eqtl-a-ENSG00000060982	7.93e-21	0.00276797886214063	87.608214511663
rs12478601	eqtl-a-ENSG00000060982	1.93e-07	0.000865072665046051	27.0984864304864
rs12578623	eqtl-a-ENSG00000060982	2.72e-37	0.00563361306967741	162.827345886415
rs12608504	eqtl-a-ENSG00000060982	3.27e-09	0.00127634439246143	35.0126955779316
rs12824095	eqtl-a-ENSG00000060982	7.17e-18	0.00242234457878449	74.1677526679895
rs12825024	eqtl-a-ENSG00000060982	5.63e-20	0.00264635047618008	83.7298140596458
rs1352627	eqtl-a-ENSG00000060982	3.61e-09	0.00109779820166181	34.8186664945113
rs138327616	eqtl-a-ENSG00000060982	7.59e-23	0.0030583615844058	96.8087928120629
rs138588701	eqtl-a-ENSG00000060982	2.32e-30	0.00445930353525297	131.117425482914
rs139157960	eqtl-a-ENSG00000060982	3.07e-06	0.00878825156011751	21.7575804185864
rs139833859	eqtl-a-ENSG00000060982	2.41e-20	0.00269860926137868	85.4148011180354
rs139924801	eqtl-a-ENSG00000060982	6.97e-08	0.00326278925865907	29.0684127216871
rs140064373	eqtl-a-ENSG00000060982	1.22e-16	0.00257062682450799	68.5677888633846
rs140475470	eqtl-a-ENSG00000060982	4.96e-06	0.00205446483233716	20.8483976568751
rs140817264	eqtl-a-ENSG00000060982	5.49e-43	0.00872725621289066	188.891789087446
rs142090834	eqtl-a-ENSG00000060982	5.76e-08	0.00404286072251884	29.4337797711361
rs142726271	eqtl-a-ENSG00000060982	1.17e-06	0.0010856209961779	23.6248768772853
rs142860917	eqtl-a-ENSG00000060982	5.52e-08	0.00166599799855467	29.520685998221

rs145220228	eqtl-a-ENSG00000060982	1.37e-06	0.00138834322891445	23.3162752850398
rs146053236	eqtl-a-ENSG00000060982	2.16e-10	0.00520082792617176	40.3027903604674
rs147920828	eqtl-a-ENSG00000060982	8.1e-19	0.00260958187407705	78.4661241789573
rs1497256	eqtl-a-ENSG00000060982	5.89e-190	0.0276372009624702	864.164547321921
rs150719195	eqtl-a-ENSG00000060982	2.8e-06	0.000724286663609128	21.9472963081026
rs151158819	eqtl-a-ENSG00000060982	2.19e-126	0.0202627747540373	571.832369678235
rs1553670	eqtl-a-ENSG00000060982	1.75e-21	0.0034277115049617	90.6033385697044
rs1553672	eqtl-a-ENSG00000060982	1.29e-44	0.00624715951204049	196.362985410215
rs17287826	eqtl-a-ENSG00000060982	2.04e-30	0.00495659942742486	131.376534152862
rs17358437	eqtl-a-ENSG00000060982	4.54e-39	0.00624182725181045	170.957139069311
rs17387444	eqtl-a-ENSG00000060982	1.41e-20	0.00273198025104459	86.4766731479409
rs182203339	eqtl-a-ENSG00000060982	2.41e-09	0.0144192389235997	35.5806347354989
rs184826564	eqtl-a-ENSG00000060982	6.47e-67	0.0386620809148318	298.570651394728
rs1877145	eqtl-a-ENSG00000060982	7.93e-07	0.000776766732680822	24.3728994271958
rs188212579	eqtl-a-ENSG00000060982	2.9e-81	0.0230753193684776	364.48588129107
rs188548728	eqtl-a-ENSG00000060982	4.15e-12	0.0117170849226287	48.0286669914282
rs2143606	eqtl-a-ENSG00000060982	5.75e-10	0.00121076957076876	38.4061024813121
rs2200509	eqtl-a-ENSG00000060982	1e-200	0.0546843754483934	1832.73219860064
rs2291438	eqtl-a-ENSG00000060982	2.84e-26	0.00355945023864987	112.43747129578
rs2291894	eqtl-a-ENSG00000060982	1e-200	0.107383879763748	3811.42128351278
rs35210171	eqtl-a-ENSG00000060982	2.36e-50	0.00700573732440656	222.646863928408
rs35679097	eqtl-a-ENSG00000060982	3.85e-13	0.0018063390586948	52.71568552

rs3923578	eqtl-a-ENSG00000060982	3.36e-15	0.0021632122959729	62.0410173777988
rs4408389	eqtl-a-ENSG00000060982	3.3e-29	0.00395637167815326	125.843651766978
rs4528424	eqtl-a-ENSG00000060982	1.35e-07	0.00089878596097747	27.7974701701434
rs554016	eqtl-a-ENSG00000060982	4.43e-08	0.00117174042353824	29.9484530856107
rs56000990	eqtl-a-ENSG00000060982	1.12e-15	0.00214778325105002	64.2041231358906
rs56024196	eqtl-a-ENSG00000060982	6.62e-21	0.00277919320119751	87.9725051772815
rs56388170	eqtl-a-ENSG00000060982	1.55e-13	0.00173498782024756	54.4916154172765
rs58322095	eqtl-a-ENSG00000060982	1.03e-87	0.0124633348078588	394.129652531831
rs61179355	eqtl-a-ENSG00000060982	6.59e-11	0.00784997073036127	42.6223767342787
rs61734770	eqtl-a-ENSG00000060982	1.17e-23	0.00344150913279636	100.511032947875
rs61761101	eqtl-a-ENSG00000060982	5.21e-21	0.00279425101359804	88.4504803395779
rs61909244	eqtl-a-ENSG00000060982	2.5e-15	0.00219008638695814	62.6246982919061
rs61909251	eqtl-a-ENSG00000060982	7.54e-16	0.00227235470861926	64.9824878084742
rs61911672	eqtl-a-ENSG00000060982	9.67e-16	0.00225530128789504	64.4937090913964
rs61911990	eqtl-a-ENSG00000060982	7.79e-18	0.00241697038385907	74.0052288212871
rs61912148	eqtl-a-ENSG00000060982	4.52e-17	0.00235080337602383	70.5324052709514
rs6487412	eqtl-a-ENSG00000060982	7e-50	0.00693800458781109	220.493207677768
rs67372216	eqtl-a-ENSG00000060982	5.91e-94	0.0134984245827784	422.808569192045
rs73069946	eqtl-a-ENSG00000060982	2.84e-35	0.00675238971111194	153.587068091784
rs73070607	eqtl-a-ENSG00000060982	4.95e-08	0.00107245659171155	29.7367940980734
rs73073207	eqtl-a-ENSG00000060982	3.37e-19	0.00252489746898895	80.196289019676
rs73281496	eqtl-a-ENSG00000060982	5.38e-08	0.000990224094050826	29.5716281606173

rs73289654	eqtl-a-ENSG00000060982	4.34e-16	0.00226251373681338	66.0723523543568
rs74629434	eqtl-a-ENSG00000060982	2.96e-16	0.00249157412820431	66.8235785513225
rs75483010	eqtl-a-ENSG00000060982	7.26e-132	0.0233112833287783	597.025880644374
rs7563201	eqtl-a-ENSG00000060982	1.3e-06	0.00074909901373116	23.4178973147161
rs75764956	eqtl-a-ENSG00000060982	1.28e-41	0.00594419551704785	182.645186571941
rs75940195	eqtl-a-ENSG00000060982	1.69e-09	0.00629941850109529	36.2864550666555
rs76889209	eqtl-a-ENSG00000060982	3.74e-06	0.000684485482495653	21.3904385775273
rs77102719	eqtl-a-ENSG00000060982	1.22e-06	0.00315124829268946	23.5320476156527
rs77190971	eqtl-a-ENSG00000060982	1.18e-37	0.00820262891936979	164.483077597474
rs77345488	eqtl-a-ENSG00000060982	1e-200	0.0370641130975479	1214.99864108369
rs77437274	eqtl-a-ENSG00000060982	1.88e-08	0.00111560798246757	31.6103174502556
rs77562188	eqtl-a-ENSG00000060982	8.53e-11	0.00146998160947801	42.1298638250743
rs77685693	eqtl-a-ENSG00000060982	6.07e-07	0.000879512740970247	24.8865927753951
rs78256655	eqtl-a-ENSG00000060982	6.84e-16	0.00346167758117205	65.1701310820979
rs78786738	eqtl-a-ENSG00000060982	2.61e-39	0.00560197809330187	172.059488410446
rs79637703	eqtl-a-ENSG00000060982	6.46e-50	0.0165040319290132	220.636402026412
rs7977807	eqtl-a-ENSG00000060982	1e-200	0.0299923998377745	975.982147687319
rs79884684	eqtl-a-ENSG00000060982	2.74e-30	0.004448199565118	130.789475357541
rs80249736	eqtl-a-ENSG00000060982	6.68e-21	0.00568445736336913	87.9439210905139
rs8070345	eqtl-a-ENSG00000060982	1.56e-06	0.000727627739046708	23.0694880278925
rs859128	eqtl-a-ENSG00000060982	4.31e-131	0.0184544850525598	593.449164942889
rs979553	eqtl-a-ENSG00000060982	5.75e-21	0.00280755899758644	88.2478903790011

rs10943133	eqtl-a-ENSG00000156535	8.76e-21	0.00275144003368368	87.4116310081316
rs11758024	eqtl-a-ENSG00000156535	1.28e-07	0.000912592374132969	27.9005959389792
rs12196481	eqtl-a-ENSG00000156535	2.18e-07	0.000878689126246711	26.862284269173
rs12211905	eqtl-a-ENSG00000156535	4.09e-34	0.00467729533483464	148.271532150318
rs12212966	eqtl-a-ENSG00000156535	6.06e-08	0.000928692091783078	29.3376967957711
rs12528608	eqtl-a-ENSG00000156535	3.53e-07	0.000817944929693009	25.9353449464327
rs13192124	eqtl-a-ENSG00000156535	2.02e-09	0.00115180373292289	35.9523468323588
rs13193410	eqtl-a-ENSG00000156535	2.86e-07	0.000862439669259215	26.3435159593131
rs13204211	eqtl-a-ENSG00000156535	7.01e-09	0.00107839918320735	33.5312386908364
rs13215297	eqtl-a-ENSG00000156535	1.17e-10	0.00130845166451144	41.5086777335235
rs13215985	eqtl-a-ENSG00000156535	5.04e-09	0.00108149210361186	34.1742574400766
rs1358880	eqtl-a-ENSG00000156535	3.1e-07	0.000825713022912158	26.1818586935903
rs145801191	eqtl-a-ENSG00000156535	8.98e-07	0.00108601930650749	24.1347533933027
rs1691348	eqtl-a-ENSG00000156535	2.18e-06	0.000710193664324659	22.432484228918
rs184668938	eqtl-a-ENSG00000156535	2.08e-09	0.00136083880225513	35.8960644864061
rs1934020	eqtl-a-ENSG00000156535	4.54e-07	0.000802659223715972	25.4470642096789
rs2351528	eqtl-a-ENSG00000156535	1.3e-34	0.00478762375156135	150.563891439149
rs2781212	eqtl-a-ENSG00000156535	7.24e-08	0.000917665690493636	28.9908861305897
rs278349	eqtl-a-ENSG00000156535	1.1e-07	0.000892487525076713	28.1965337836988
rs2882704	eqtl-a-ENSG00000156535	1.38e-20	0.00272350837754711	86.5218353608935
rs34906464	eqtl-a-ENSG00000156535	3.26e-10	0.00126576869531107	39.5128847863372
rs4301271	eqtl-a-ENSG00000156535	1.21e-19	0.00259859123617	82.227814482846

rs4708088	eqtl-a-ENSG00000156535	1.79e-33	0.00458431267532878	145.365646861542
rs4708093	eqtl-a-ENSG00000156535	3e-19	0.00253236645145942	80.4341225887335
rs562131	eqtl-a-ENSG00000156535	2.33e-06	0.000705941825250617	22.2995018068687
rs62440612	eqtl-a-ENSG00000156535	3.44e-08	0.00119746889324688	30.4353907576514
rs686017	eqtl-a-ENSG00000156535	2.94e-07	0.000829090671816933	26.2890466678674
rs6924739	eqtl-a-ENSG00000156535	2.43e-41	0.00571428543818185	181.356313025913
rs72950536	eqtl-a-ENSG00000156535	1.04e-09	0.0013537995691504	37.2528450458256
rs72958825	eqtl-a-ENSG00000156535	7.54e-08	0.000946105880699783	28.9204893357239
rs7449500	eqtl-a-ENSG00000156535	3.17e-06	0.000689507276150605	21.7151457460064
rs7453832	eqtl-a-ENSG00000156535	2.79e-10	0.00142907861990023	39.8152954717479
rs7770133	eqtl-a-ENSG00000156535	6.31e-13	0.00192860822250766	51.7440650115575
rs7771585	eqtl-a-ENSG00000156535	1.2e-35	0.00487773008015144	155.288823635436
rs7774119	eqtl-a-ENSG00000156535	1.71e-25	0.00342587267470603	108.89098950947
rs78258948	eqtl-a-ENSG00000156535	1.65e-07	0.00108949922645529	27.4046148706185
rs79717953	eqtl-a-ENSG00000156535	4.22e-34	0.0260144715063616	148.183195432597
rs9341441	eqtl-a-ENSG00000156535	4.82e-08	0.000943200346864537	29.7898415338313
rs9447087	eqtl-a-ENSG00000156535	6.15e-07	0.000787157438437819	24.8677737122216
rs11960421	eqtl-a-ENSG00000164309	1.78e-07	0.000903979605887773	27.2606446125658
rs16877016	eqtl-a-ENSG00000164309	1.73e-10	0.00137360733524887	40.7504660531685
rs1948763	eqtl-a-ENSG00000164309	4.33e-11	0.00146460852743134	43.4585358013875
rs259116	eqtl-a-ENSG00000164309	3.16e-06	0.000732523407052852	21.7169140818346
rs3736401	eqtl-a-ENSG00000164309	4.21e-11	0.00146087537312101	43.5158483292759

rs6866361	eqtl-a-ENSG00000164309	2.71e-11	0.00149575751300249	44.3781953417947
rs6875026	eqtl-a-ENSG00000164309	2.27e-10	0.0013556473036266	40.2209994486042
rs7707820	eqtl-a-ENSG00000164309	1.6e-11	0.00152425108016112	45.4019553545626
rs10100356	eqtl-a-ENSG00000163751	8.83e-07	0.000770157403946257	24.1653562139341
rs112875651	eqtl-a-ENSG00000163751	2.16e-06	0.000718083376839553	22.4476077797104
rs113892033	eqtl-a-ENSG00000163751	2.19e-23	0.00323996343686558	99.2798663893035
rs115004392	eqtl-a-ENSG00000163751	1.62e-08	0.00123728013158281	31.9006707344728
rs115569504	eqtl-a-ENSG00000163751	2.69e-09	0.00137244744078651	35.3890912679951
rs116344051	eqtl-a-ENSG00000163751	3.93e-08	0.00116205023874239	30.1833059117734
rs116617835	eqtl-a-ENSG00000163751	9.89e-08	0.00110203896893349	28.3944687545109
rs12107440	eqtl-a-ENSG00000163751	8.55e-32	0.00432652865579625	137.668708485204
rs13233571	eqtl-a-ENSG00000163751	2.28e-07	0.000855773799390502	26.7812538547059
rs149324937	eqtl-a-ENSG00000163751	4.79e-14	0.00218645584011768	56.8080760045969
rs1546632	eqtl-a-ENSG00000163751	3.93e-36	0.0049477774676336	157.529961144327
rs16823866	eqtl-a-ENSG00000163751	2.77e-07	0.000832679396721816	26.4029338259504
rs16860983	eqtl-a-ENSG00000163751	8.21e-07	0.000779492234975027	24.3055855803433
rs16861212	eqtl-a-ENSG00000163751	1.71e-25	0.00349374879818148	108.892787969819
rs17145738	eqtl-a-ENSG00000163751	1.15e-06	0.000763376193383662	23.6590580946059
rs2035403	eqtl-a-ENSG00000163751	1.32e-13	0.00173368879662914	54.8223992225286
rs340630	eqtl-a-ENSG00000163751	6.05e-07	0.000794784732784446	24.8949586997855
rs34197730	eqtl-a-ENSG00000163751	1.38e-06	0.000745530849184411	23.3062682087516
rs36098024	eqtl-a-ENSG00000163751	5.42e-09	0.00131004755267438	34.032457844695

rs3772578	eqtl-a-ENSG00000163751	1.19e-38	0.0053081387282937	169.069897661366
rs3772592	eqtl-a-ENSG00000163751	3.1e-20	0.002673017080206	84.9135023772809
rs4328821	eqtl-a-ENSG00000163751	6.23e-32	0.00441568337720228	138.304965689277
rs442177	eqtl-a-ENSG00000163751	7.28e-10	0.00121541387311435	37.9463713514996
rs4857855	eqtl-a-ENSG00000163751	1.84e-16	0.0021601841137839	67.7558074119967
rs4982731	eqtl-a-ENSG00000163751	7.38e-41	0.00562302759664766	179.15050552592
rs514406	eqtl-a-ENSG00000163751	3.36e-09	0.00110630986357504	34.9604542489803
rs55779678	eqtl-a-ENSG00000163751	2.46e-09	0.00150082729186733	35.5614444847101
rs58612888	eqtl-a-ENSG00000163751	2.52e-06	0.000774195061976376	22.1482871361888
rs6058796	eqtl-a-ENSG00000163751	7.01e-07	0.000779039639379587	24.6111173322667
rs6141743	eqtl-a-ENSG00000163751	1.49e-06	0.000733023448006384	23.1563253126414
rs61925898	eqtl-a-ENSG00000163751	3.96e-10	0.00125421532171922	39.1216368738968
rs62275033	eqtl-a-ENSG00000163751	4.16e-09	0.00123657572470148	34.544416081246
rs651821	eqtl-a-ENSG00000163751	9.95e-07	0.000754857989400437	23.9334771966607
rs6568466	eqtl-a-ENSG00000163751	3.02e-06	0.000699710052749157	21.8035274799191
rs6766148	eqtl-a-ENSG00000163751	4.86e-39	0.00538242033963045	170.826321930819
rs6785206	eqtl-a-ENSG00000163751	9.52e-28	0.00378679010001996	119.178534098987
rs6840258	eqtl-a-ENSG00000163751	9.22e-08	0.000921894675380536	28.5275952222014
rs714052	eqtl-a-ENSG00000163751	5.04e-08	0.000949837221725412	29.699224561203
rs72725119	eqtl-a-ENSG00000163751	3.74e-07	0.000817227591467806	25.818522989222
rs74455390	eqtl-a-ENSG00000163751	2.15e-29	0.00413096587606757	126.703761600023
rs7648905	eqtl-a-ENSG00000163751	3.92e-46	0.00640162425251561	203.324062409931

rs77076342	eqtl-a-ENSG00000163751	6.13e-27	0.00367006658834543	115.491459089638
rs78744187	eqtl-a-ENSG00000163751	3.53e-24	0.00433005811802918	102.886017391171
rs79450911	eqtl-a-ENSG00000163751	7.57e-08	0.00115324261035687	28.9116903992522
rs8022206	eqtl-a-ENSG00000163751	3.51e-08	0.000962098333783789	30.399805704969
rs964184	eqtl-a-ENSG00000163751	2.25e-07	0.000855720080127291	26.8052648712274
rs9880192	eqtl-a-ENSG00000163751	1.58e-06	0.00072968170655164	23.0506820917615
rs11789820	eqtl-a-ENSG00000136943	2.24e-07	0.00124955027757417	26.8101134828811
rs12684560	eqtl-a-ENSG00000136943	4.1e-07	0.00123008022024636	25.6418116708768
rs16911041	eqtl-a-ENSG00000136943	3.35e-06	0.000891995460281994	21.6019910349193
rs62558840	eqtl-a-ENSG00000136943	2.03e-06	0.00106254366921309	22.5658411339932
rs62558846	eqtl-a-ENSG00000136943	5.79e-07	0.0011761350363977	24.981085927584
rs72747714	eqtl-a-ENSG00000136943	1.44e-07	0.00129277404096415	27.6623424135883
rs75837839	eqtl-a-ENSG00000136943	1.75e-06	0.00107383499933986	22.8478238688985

Table S4 CRSwNP tool variable information

SNP	id	pvalue	r2	F
rs10943133	eqtl-a-ENSG00000156535	8.76e-21	0.00275144003368368	87.4116310081316
rs11758024	eqtl-a-ENSG00000156535	1.28e-07	0.000912592374132969	27.9005959389792
rs12196481	eqtl-a-ENSG00000156535	2.18e-07	0.000878689126246711	26.862284269173
rs12211905	eqtl-a-ENSG00000156535	4.09e-34	0.00467729533483464	148.271532150318
rs12212966	eqtl-a-ENSG00000156535	6.06e-08	0.000928692091783078	29.3376967957711
rs12528608	eqtl-a-ENSG00000156535	3.53e-07	0.000817944929693009	25.9353449464327
rs13192124	eqtl-a-ENSG00000156535	2.02e-09	0.00115180373292289	35.9523468323588
rs13193410	eqtl-a-ENSG00000156535	2.86e-07	0.000862439669259215	26.3435159593131
rs13215297	eqtl-a-ENSG00000156535	1.17e-10	0.00130845166451144	41.5086777335235
rs13215985	eqtl-a-ENSG00000156535	5.04e-09	0.00108149210361186	34.1742574400766
rs1358880	eqtl-a-ENSG00000156535	3.1e-07	0.000825713022912158	26.1818586935903
rs145801191	eqtl-a-ENSG00000156535	8.98e-07	0.00108601930650749	24.1347533933027
rs1691348	eqtl-a-ENSG00000156535	2.18e-06	0.000710193664324659	22.432484228918
rs184668938	eqtl-a-ENSG00000156535	2.08e-09	0.00136083880225513	35.8960644864061
rs2351528	eqtl-a-ENSG00000156535	1.3e-34	0.00478762375156135	150.563891439149
rs2781212	eqtl-a-ENSG00000156535	7.24e-08	0.000917665690493636	28.9908861305897
rs278349	eqtl-a-ENSG00000156535	1.1e-07	0.000892487525076713	28.1965337836988
rs2882704	eqtl-a-ENSG00000156535	1.38e-20	0.00272350837754711	86.5218353608935
rs4301271	eqtl-a-ENSG00000156535	1.21e-19	0.00259859123617	82.227814482846
rs4708088	eqtl-a-ENSG00000156535	1.79e-33	0.00458431267532878	145.365646861542
rs4708093	eqtl-a-ENSG00000156535	3e-19	0.00253236645145942	80.4341225887335

rs562131	eqtl-a-ENSG00000156535	2.33e-06	0.000705941825250617	22.2995018068687
rs61840520	eqtl-a-ENSG00000156535	1e-54	0.00763110889015438	242.720547940041
rs62440612	eqtl-a-ENSG00000156535	3.44e-08	0.00119746889324688	30.4353907576514
rs6924739	eqtl-a-ENSG00000156535	2.43e-41	0.00571428543818185	181.356313025913
rs72950536	eqtl-a-ENSG00000156535	1.04e-09	0.0013537995691504	37.2528450458256
rs72958825	eqtl-a-ENSG00000156535	7.54e-08	0.000946105880699783	28.9204893357239
rs7453832	eqtl-a-ENSG00000156535	2.79e-10	0.00142907861990023	39.8152954717479
rs74934634	eqtl-a-ENSG00000156535	1.47e-06	0.00103233581388724	23.1823212534114
rs7770133	eqtl-a-ENSG00000156535	6.31e-13	0.00192860822250766	51.7440650115575
rs7771585	eqtl-a-ENSG00000156535	1.2e-35	0.00487773008015144	155.288823635436
rs7774119	eqtl-a-ENSG00000156535	1.71e-25	0.00342587267470603	108.89098950947
rs78258948	eqtl-a-ENSG00000156535	1.65e-07	0.00108949922645529	27.4046148706185
rs79717953	eqtl-a-ENSG00000156535	4.22e-34	0.0260144715063616	148.183195432597
rs9341441	eqtl-a-ENSG00000156535	4.82e-08	0.000943200346864537	29.7898415338313
rs10100356	eqtl-a-ENSG00000163751	8.83e-07	0.000770157403946257	24.1653562139341
rs112875651	eqtl-a-ENSG00000163751	2.16e-06	0.000718083376839553	22.4476077797104
rs113892033	eqtl-a-ENSG00000163751	2.19e-23	0.00323996343686558	99.2798663893035
rs115004392	eqtl-a-ENSG00000163751	1.62e-08	0.00123728013158281	31.9006707344728
rs115569504	eqtl-a-ENSG00000163751	2.69e-09	0.00137244744078651	35.3890912679951
rs116344051	eqtl-a-ENSG00000163751	3.93e-08	0.00116205023874239	30.1833059117734
rs116617835	eqtl-a-ENSG00000163751	9.89e-08	0.00110203896893349	28.3944687545109
rs12107440	eqtl-a-ENSG00000163751	8.55e-32	0.00432652865579625	137.668708485204

rs13233571	eqtl-a-ENSG00000163751	2.28e-07	0.000855773799390502	26.7812538547059
rs149324937	eqtl-a-ENSG00000163751	4.79e-14	0.00218645584011768	56.8080760045969
rs1546632	eqtl-a-ENSG00000163751	3.93e-36	0.0049477774676336	157.529961144327
rs16823866	eqtl-a-ENSG00000163751	2.77e-07	0.000832679396721816	26.4029338259504
rs16860983	eqtl-a-ENSG00000163751	8.21e-07	0.000779492234975027	24.3055855803433
rs16861212	eqtl-a-ENSG00000163751	1.71e-25	0.00349374879818148	108.892787969819
rs17145738	eqtl-a-ENSG00000163751	1.15e-06	0.000763376193383662	23.6590580946059
rs2035403	eqtl-a-ENSG00000163751	1.32e-13	0.00173368879662914	54.8223992225286
rs340630	eqtl-a-ENSG00000163751	6.05e-07	0.000794784732784446	24.8949586997855
rs34197730	eqtl-a-ENSG00000163751	1.38e-06	0.000745530849184411	23.3062682087516
rs36098024	eqtl-a-ENSG00000163751	5.42e-09	0.00131004755267438	34.032457844695
rs3772578	eqtl-a-ENSG00000163751	1.19e-38	0.0053081387282937	169.069897661366
rs3772592	eqtl-a-ENSG00000163751	3.1e-20	0.002673017080206	84.9135023772809
rs4328821	eqtl-a-ENSG00000163751	6.23e-32	0.00441568337720228	138.304965689277
rs442177	eqtl-a-ENSG00000163751	7.28e-10	0.00121541387311435	37.9463713514996
rs4857855	eqtl-a-ENSG00000163751	1.84e-16	0.0021601841137839	67.7558074119967
rs4982731	eqtl-a-ENSG00000163751	7.38e-41	0.00562302759664766	179.15050552592
rs514406	eqtl-a-ENSG00000163751	3.36e-09	0.00110630986357504	34.9604542489803
rs55779678	eqtl-a-ENSG00000163751	2.46e-09	0.00150082729186733	35.5614444847101
rs58612888	eqtl-a-ENSG00000163751	2.52e-06	0.000774195061976376	22.1482871361888
rs6058796	eqtl-a-ENSG00000163751	7.01e-07	0.000779039639379587	24.6111173322667
rs61925898	eqtl-a-ENSG00000163751	3.96e-10	0.00125421532171922	39.1216368738968

rs62275033	eqtl-a-ENSG00000163751	4.16e-09	0.00123657572470148	34.544416081246
rs651821	eqtl-a-ENSG00000163751	9.95e-07	0.000754857989400437	23.9334771966607
rs6568466	eqtl-a-ENSG00000163751	3.02e-06	0.000699710052749157	21.8035274799191
rs6766148	eqtl-a-ENSG00000163751	4.86e-39	0.00538242033963045	170.826321930819
rs6785206	eqtl-a-ENSG00000163751	9.52e-28	0.00378679010001996	119.178534098987
rs6840258	eqtl-a-ENSG00000163751	9.22e-08	0.000921894675380536	28.5275952222014
rs714052	eqtl-a-ENSG00000163751	5.04e-08	0.000949837221725412	29.699224561203
rs72725119	eqtl-a-ENSG00000163751	3.74e-07	0.000817227591467806	25.818522989222
rs74455390	eqtl-a-ENSG00000163751	2.15e-29	0.00413096587606757	126.703761600023
rs7648905	eqtl-a-ENSG00000163751	3.92e-46	0.00640162425251561	203.324062409931
rs77076342	eqtl-a-ENSG00000163751	6.13e-27	0.00367006658834543	115.491459089638
rs78744187	eqtl-a-ENSG00000163751	3.53e-24	0.00433005811802918	102.886017391171
rs79450911	eqtl-a-ENSG00000163751	7.57e-08	0.00115324261035687	28.9116903992522
rs8022206	eqtl-a-ENSG00000163751	3.51e-08	0.000962098333783789	30.399805704969
rs11789820	eqtl-a-ENSG00000136943	2.24e-07	0.00124955027757417	26.8101134828811
rs12684560	eqtl-a-ENSG00000136943	4.1e-07	0.00123008022024636	25.6418116708768
rs16911041	eqtl-a-ENSG00000136943	3.35e-06	0.000891995460281994	21.6019910349193
rs62558840	eqtl-a-ENSG00000136943	2.03e-06	0.00106254366921309	22.5658411339932
rs62558846	eqtl-a-ENSG00000136943	5.79e-07	0.0011761350363977	24.981085927584
rs72747714	eqtl-a-ENSG00000136943	1.44e-07	0.00129277404096415	27.6623424135883
rs75837839	eqtl-a-ENSG00000136943	1.75e-06	0.00107383499933986	22.8478238688985
rs11119332	eqtl-a-ENSG00000196878	7.6e-32	0.00435014806665574	137.90799376839

rs11119399	eqtl-a-ENSG00000196878	2.06e-06	0.000713566176781295	22.5369437061512
rs12030655	eqtl-a-ENSG00000196878	8.2e-37	0.00552412524989586	160.61735197033
rs12086634	eqtl-a-ENSG00000196878	2.27e-33	0.00460771383792993	144.879792323456
rs12130989	eqtl-a-ENSG00000196878	2.61e-06	0.000696553798664209	22.0835998646534
rs2046850	eqtl-a-ENSG00000196878	7.06e-07	0.000775697652848249	24.5947311127348
rs2049998	eqtl-a-ENSG00000196878	3.46e-15	0.00195308904336044	61.9851579813461
rs2228339	eqtl-a-ENSG00000196878	1.88e-11	0.00171321603924607	45.0971522438568
rs35898529	eqtl-a-ENSG00000196878	3.46e-10	0.00124643135649508	39.3902100980593
rs3766612	eqtl-a-ENSG00000196878	1.04e-19	0.0026078785610668	82.5276932248066
rs61820640	eqtl-a-ENSG00000196878	1.43e-10	0.00129605065876902	41.1147637878124
rs642961	eqtl-a-ENSG00000196878	8.03e-16	0.00206809821815521	64.8594055546618
rs661849	eqtl-a-ENSG00000196878	9.62e-18	0.00231724373574204	73.5854314157693
rs6752	eqtl-a-ENSG00000196878	9.99e-34	0.00465948361187496	146.515203273004
rs680331	eqtl-a-ENSG00000196878	7.55e-21	0.00276130678087958	87.7148839084239
rs78075213	eqtl-a-ENSG00000196878	1.95e-06	0.00110332598928991	22.6453746628245
rs10736122	eqtl-a-ENSG00000138131	1.5e-11	0.00148013432707397	45.5341629366753
rs10748715	eqtl-a-ENSG00000138131	4.04e-10	0.0012659933928986	39.0851217825729
rs10748719	eqtl-a-ENSG00000138131	2.21e-11	0.00145533764258092	44.7716749463141
rs10786380	eqtl-a-ENSG00000138131	3.74e-06	0.000693345594291726	21.3913696294967
rs11189472	eqtl-a-ENSG00000138131	3.13e-06	0.000707265323401249	21.7340753088077
rs11189478	eqtl-a-ENSG00000138131	9.2e-07	0.000783575836666707	24.0840527775134
rs1983865	eqtl-a-ENSG00000138131	5.84e-12	0.00153435820304285	47.3831034861429

rs4919185	eqtl-a-ENSG0000013 8131	3.93e-10	0.00126811040880843	39.1505635823888
rs7084747	eqtl-a-ENSG0000013 8131	1.36e-11	0.00148657829736495	45.7282309993449
rs7899632	eqtl-a-ENSG0000013 8131	1.34e-11	0.00148149624099782	45.7482309270776
rs881990	eqtl-a-ENSG0000013 8131	9.21e-11	0.00136471971675494	41.9760163169434
rs928578	eqtl-a-ENSG0000013 8131	2.89e-06	0.000709420359649829	21.8897964367036
rs928579	eqtl-a-ENSG0000013 8131	1.52e-11	0.00147383417087665	45.5068508741252
rs9419846	eqtl-a-ENSG0000013 8131	4.98e-10	0.00125749074833238	38.6774948900152

Table S5 Correlation analysis results between key genes and immune cells in AR

Gene	Cell	Correlation	Pvalue
CD109	CD56bright natural killer cell	0.417692307692308	0.0377461388317959
CPA3	CD56bright natural killer cell	0.626923076923077	0.000797554156457317
CD109	Central memory CD8 T cell	0.31	0.131533842242238
CPA3	Central memory CD8 T cell	0.425384615384615	0.0340130010967161

Table S6 Correlation analysis results between key genes and immune cells in CRSwNP

Gene	gene	Correlation	Pvalue
CPA3	Activated B cell	0.423532499343889	0.000258919692116695
CD109	Activated B cell	-0.165252384	0.171585804224102
CPA3	Activated CD4 T cell	0.332691802991864	0.00489262310552719
CD109	Activated CD4 T cell	0.173265681042778	0.151453238114896
CPA3	Activated CD8 T cell	0.553564867465664	6.68070570665147e-07
CD109	Activated CD8 T cell	-0.094357449	0.437175146104722
CPA3	Activated dendritic cell	0.622675181523926	8.64394747434851e-09
CD109	Activated dendritic cell	0.0716822675181524	0.55539340893882
CPA3	CD56bright natural killer cell	0.565112413612107	3.45903627943878e-07
CD109	CD56bright natural killer cell	0.518222377744729	4.3273429407715e-06
CPA3	Central memory CD4 T cell	0.557274079258158	5.42216614857625e-07
CD109	Central memory CD4 T cell	0.10513515877876	0.38638617976856
CPA3	Central memory CD8 T cell	0.272154667133234	0.0226541898086234
CD109	Central memory CD8 T cell	0.267255708162016	0.0253116244333415
CPA3	Effector memeory CD4 T cell	0.508074534161491	7.12498439886243e-06
CD109	Effector memeory CD4 T cell	0.174630391041904	0.148207626562431
CPA3	Effector memeory CD8 T cell	0.497191846732569	1.19523680046446e-05
CD109	Effector memeory CD8 T cell	-0.038369347	0.752490661792522
CPA3	Gamma delta T cell	0.534388942349751	1.89002009802337e-06
CD109	Gamma delta T cell	0.48431458315108	2.1556622727982e-05
CPA3	Immature B cell	0.492572828273992	1.48086409122388e-05
CD109	Immature B cell	-0.098451579	0.417454002827884
CPA3	Immature dendritic cell	0.49992126673082	1.05154063107358e-05
CD109	Immature dendritic cell	0.475496457002887	3.18509172929571e-05
CPA3	MDSC	0.631318344851719	4.64934355267677e-09
CD109	MDSC	0.180999037704488	0.133745243792154
CPA3	Macrophage	0.650844195608433	1.06462461122365e-09

CD109	Macrophage	0.211477561018284	0.0788452783470004
CPA3	Mast cell	0.462304260344677	5.6005259931225e-05
CD109	Mast cell	0.134004024144869	0.268740411581465
CPA3	Memory B cell	0.232403114338203	0.0528663451294683
CD109	Memory B cell	0.331781996325781	0.00501830786404047
CPA3	Monocyte	0.474131747003762	3.38023341270361e-05
CD109	Monocyte	0.080010497769224	0.510268700066489
CPA3	Natural killer T cell	0.360965794768612	0.00214134324389945
CD109	Natural killer T cell	0.187822587700114	0.119462338480967
CPA3	Natural killer cell	0.370098853993526	0.00161324802293379
CD109	Natural killer cell	-0.019123436	0.875141132521423
CPA3	Neutrophil	0.348158516315283	0.00314238157989436
CD109	Neutrophil	0.28692152917505	0.0160311968684101
CPA3	Regulatory T cell	0.622185285626804	8.94811102008958e-09
CD109	Regulatory T cell	0.192581576415012	0.110213975739514
CPA3	T follicular helper cell	0.489913393403902	1.6729703924938e-05
CD109	T follicular helper cell	0.00659609832910507	0.956780751396926
CPA3	Type 2 T helper cell	-0.490473274	1.63069464441574e-05
CD109	Type 2 T helper cell	-0.202099554	0.0933857146491022
