# Supplementary material for: Key genes associated with the comorbidity of allergic rhinitis and chronic rhinosinusitis with nasal polyps: Identification, experimental validation, and an observational study using transcriptomic profiling and Mendelian randomization analysis in humans
Source: Medicine (Baltimore). 2025 Nov 28;104(48):e45983. doi: 10.1097/MD.0000000000045983 (PMC12662326; doi:10.1097/MD.0000000000045983)

Figure S1 The scatter plots of candidate genes related to AR.

Scatter plot of BCAT1 in AR(A),scatter plot of CMYA5 in AR(B), scatter plot of CPA3 in AR(C), scatter plot of CTSV in AR(D).

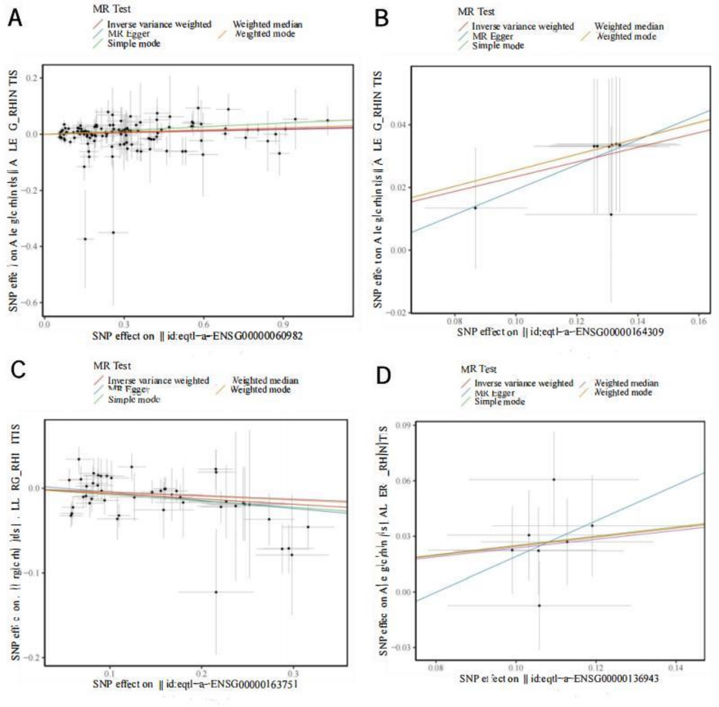

Figure S2 The forest plots plots of candidate genes related to AR.

Forest plot of BCAT1 in AR(A),forest plot of CMYA5 in AR(B), forest plot of CPA3 in AR(C), forest plot of CTSV in AR(D).

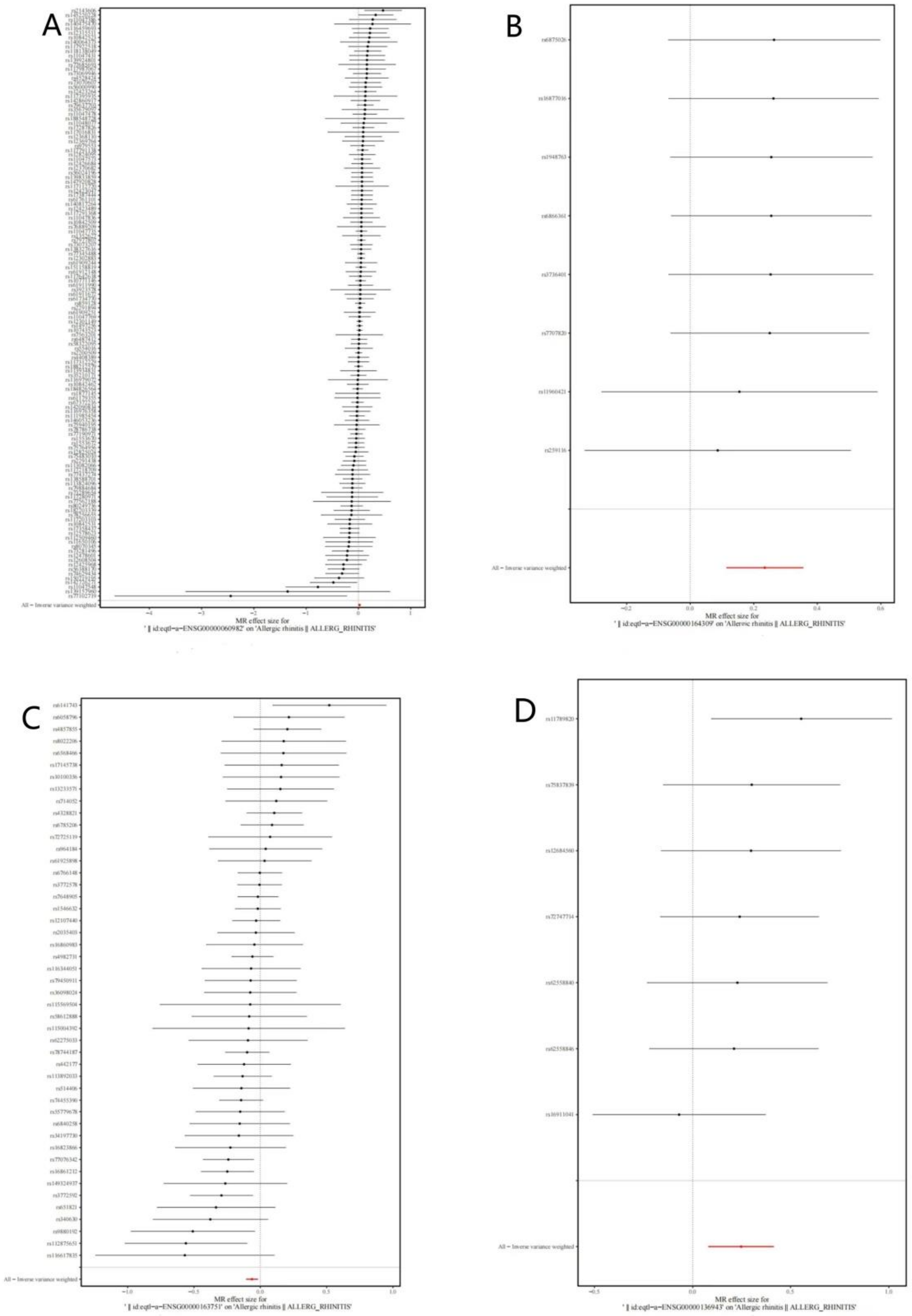

Figure S3 The funnel plots plots of candidate genes related to AR.

Funnel plot of BCAT1 in AR(A),funnel plot of CMYA5 in AR(B), funnel plot of CPA3 in AR(C), funnel plot of CTSV in AR(D).

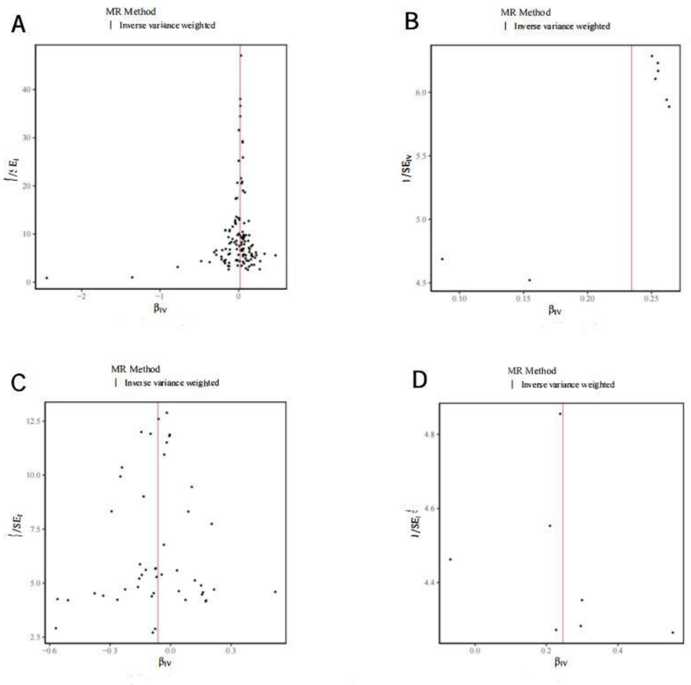

Figure S4 The scatter plots of candidate genes related to CRSwNP.

Scatter plot of CPA3 in CRSwNP(A),scatter plot of CTSV in CRSwNP(B), scatter plot of LAMB3 in CRSwNP(C), scatter plot of LOXL4 in CRSwNP(D).

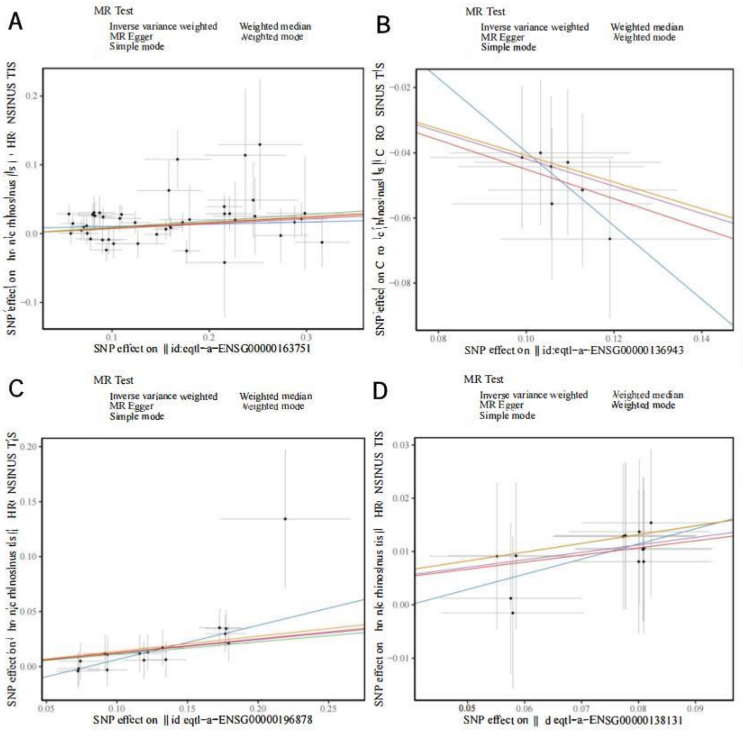

Figure S5 The forest plots of candidate genes related to CRSwNP.

Forest plot of CPA3 in CRSwNP(A),forest plot of CTSV in CRSwNP(B), forest plot of LAMB3 in CRSwNP(C), forest plot of LOXL4 in CRSwNP(D).

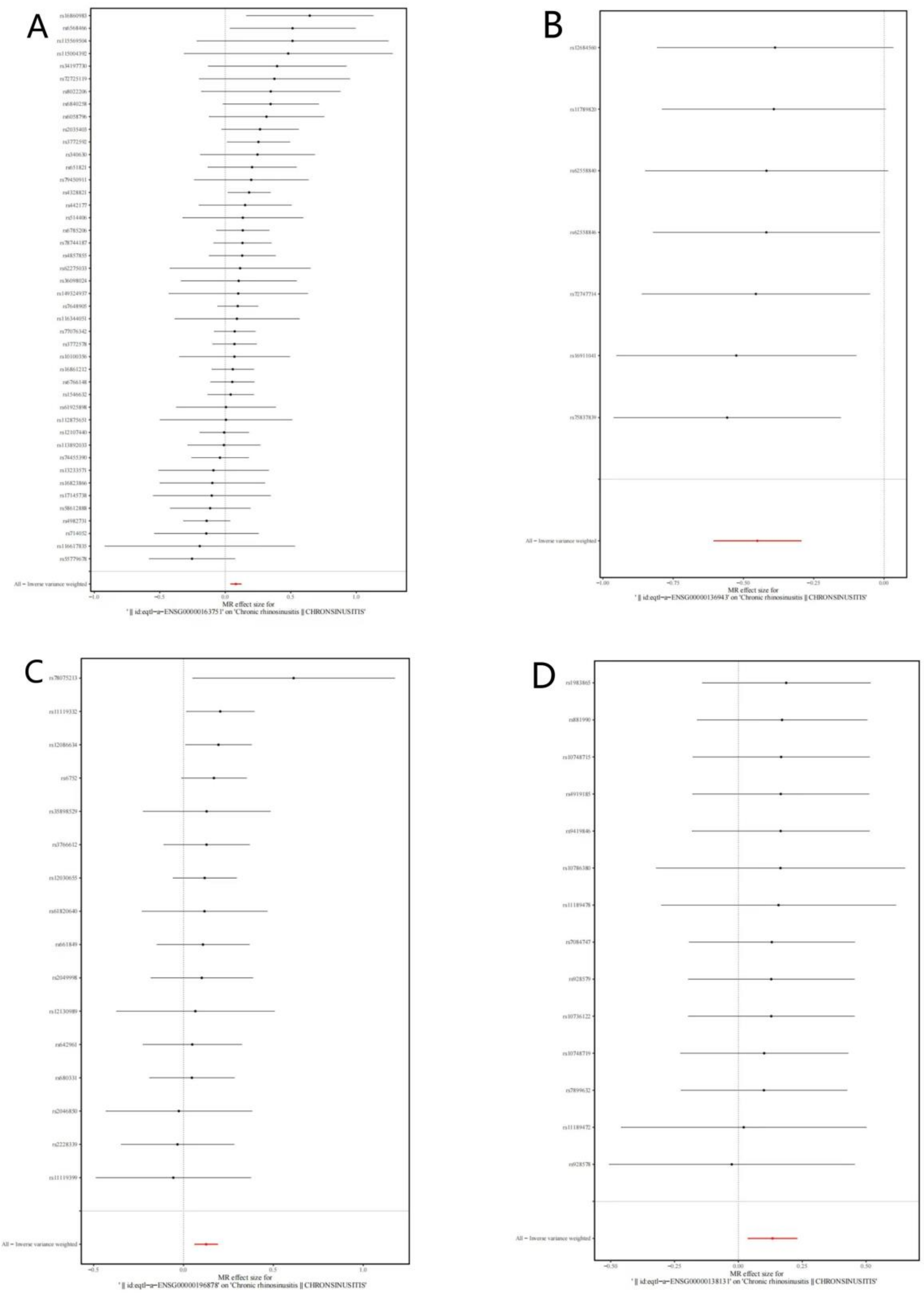

Figure S6 The funnelplots of candidate genes related to CRSwNP

Funnel plot of CPA3 in CRSwNP(A),funnel plot of CTSV in CRSwNP(B), funnel plot of LAMB3 in CRSwNP(C), funnel plot of LOXL4 in CRSwNP(D).

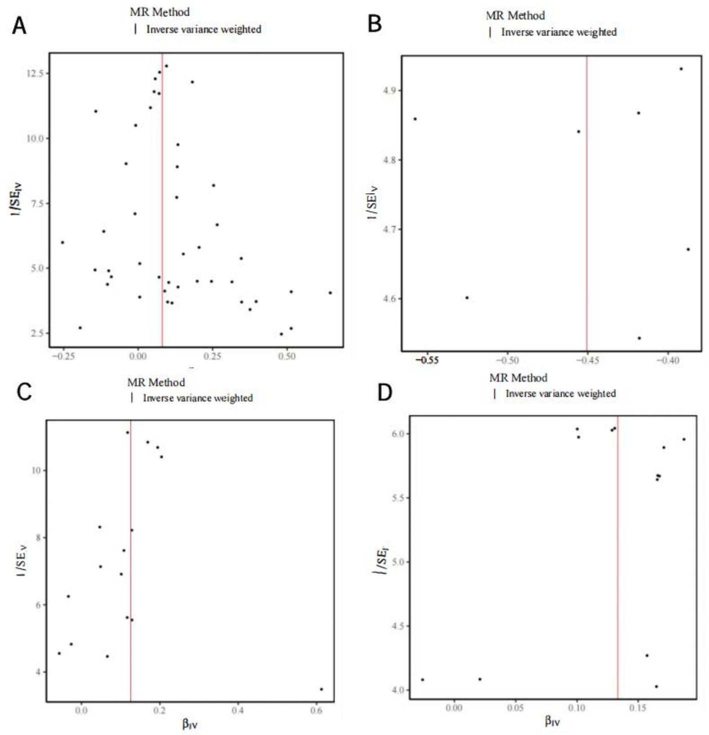

Figure S7 One by one cull test presentation of MR analysis in AR.

MR leave−one−out sensitivity analysis for BCAT1(A)、CMYA5(B)、CPA3(C)、CTSV(D) on AR.

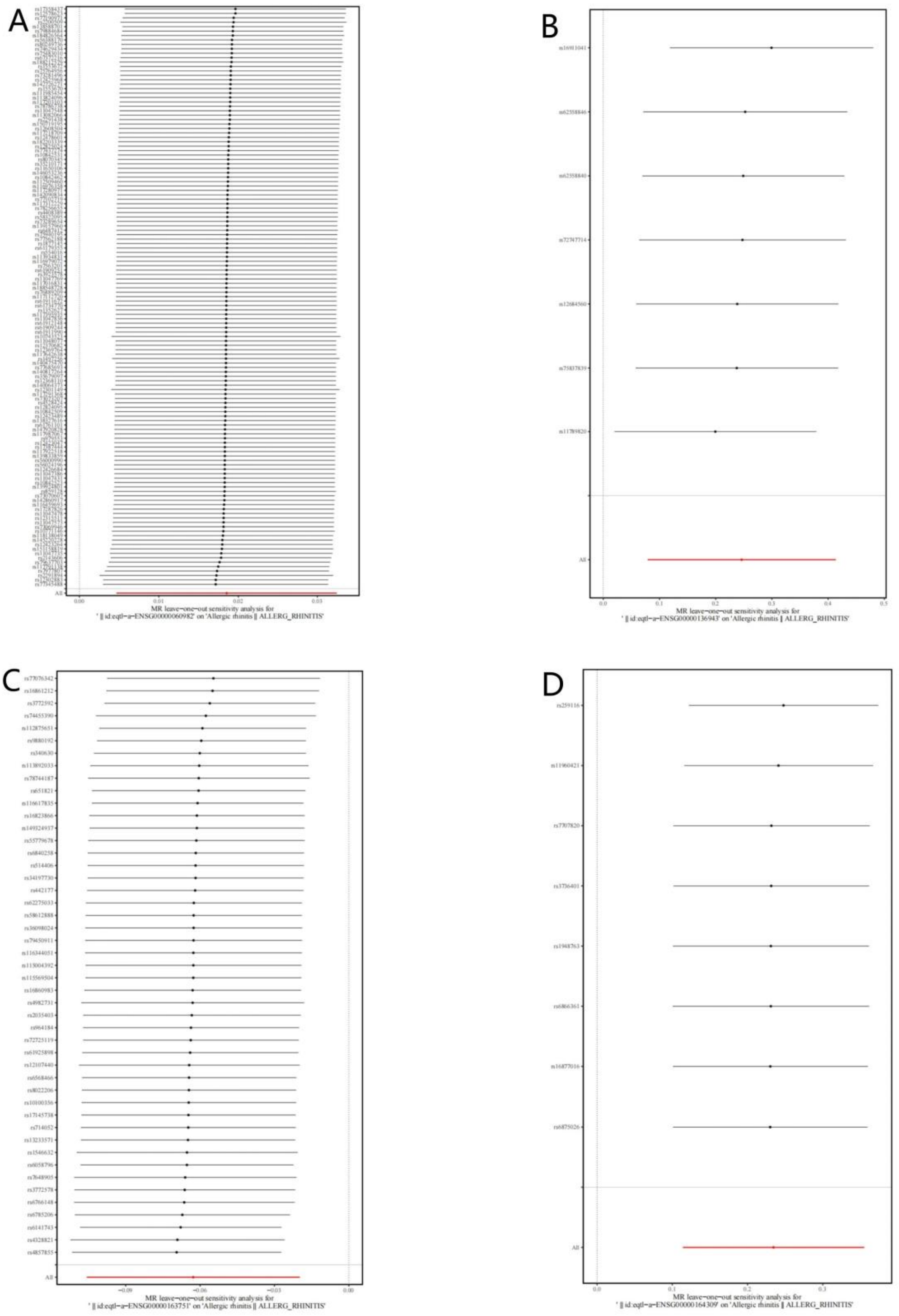

Figure S8 One by one cull test presentation of MR analysis in CRSwNP.

MR leave−one−out sensitivity analysis for CPA3(A)、CTSV(B)、LAMB3(C)、LOXL4(D) on CRSwNP.

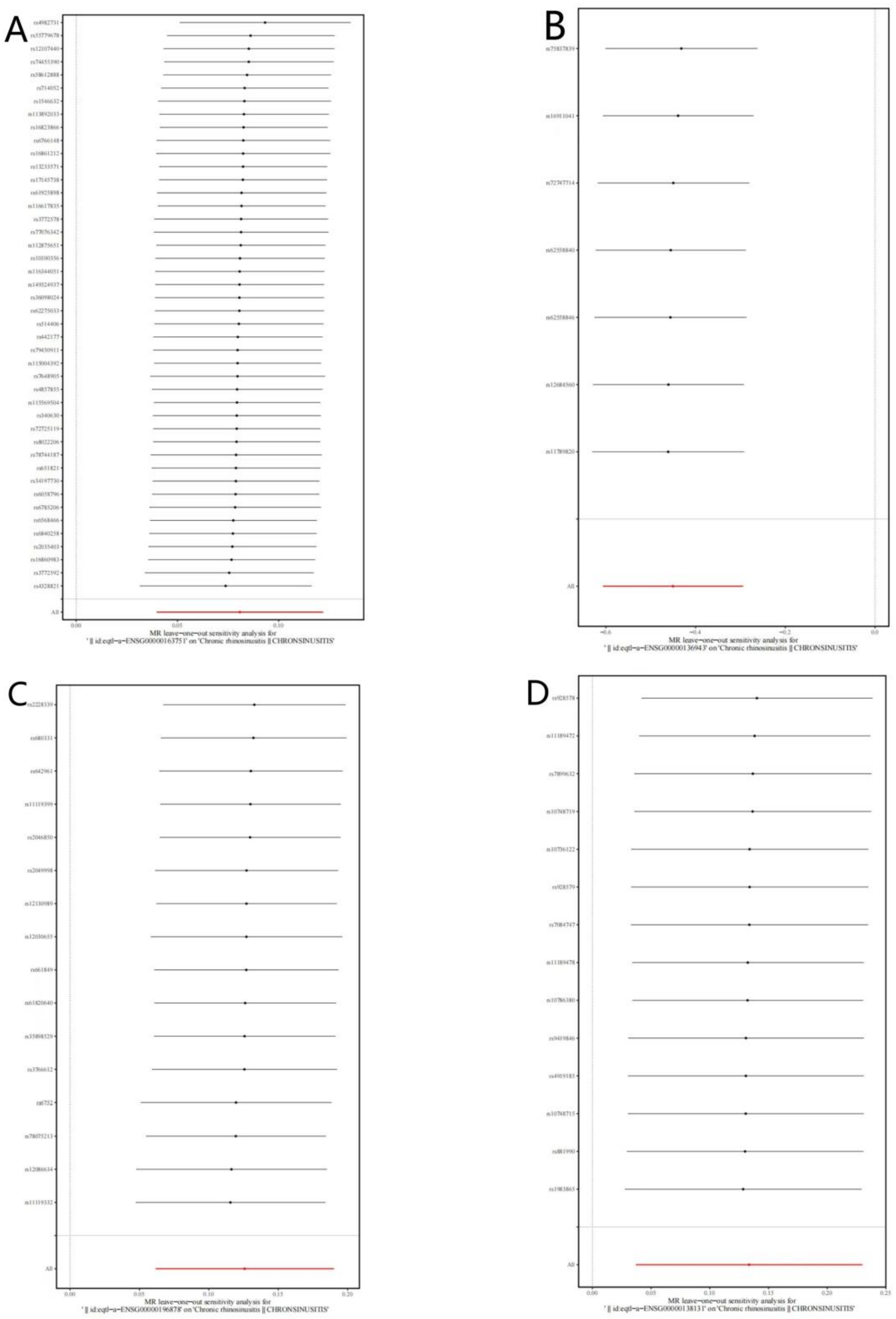

Supplement: Supplementary file 2 [file medi-104-e45983-s002.pdf]
